# Resilience and adolescence-transition in youth with developmental disabilities and their families: a scoping review

**DOI:** 10.3389/fresc.2024.1341740

**Published:** 2024-02-27

**Authors:** Naomi Zukerman, Emily Bottone, Maya Low, Tatiana Ogourtsova

**Affiliations:** ^1^ School of Physical and Occupational Therapy, Faculty of Medicine and Health Sciences, McGill University, Montreal, QC, Canada; ^2^ Faculty of Medicine and Health Sciences, McGill University, Montreal, QC, Canada; ^3^ The Research Center of the Jewish Rehabilitation Hospital, Centre Intégré de Santé et de Services Sociaux de Laval, Laval, QC, Canada; ^4^ Centre for Interdisciplinary Research in Rehabilitation of Greater Montreal, Montreal, QC, Canada

**Keywords:** resilience, adolescents with developmental disabilities, caregivers, wellbeing, scoping review, youth mental health

## Abstract

**Background:**

Children with neurodevelopmental disabilities (NDDs, e.g., cerebral palsy) and their caregivers face lifelong and impactful challenges, particularly during life-transition periods such as adolescence. One's resilience emerges as an essential ability to navigate this vulnerable phase. Resilience is a complex concept that embeds multiple factors on various levels. Little is known about what resilience factors are pivotal in youth with NDDs and their families as they transition into adolescence and how these are addressed as part of existing targeted interventions.

**Objectives:**

This review explored the concept of resilience in youth with NDDs and their families. Specific aims included describing salient resilience factors in adolescents with NDDs and their families and to describe how resilience is addressed as part of targeted interventions.

**Methods:**

Using the Arskey and O'Malley framework, six steps were undertaken, including a comprehensive literature search (*n* = 5 databases), transparent study selection, detailed data extraction with a coding scheme (*n* = 46 factors), results' collating with numerical and inductive content analysis, and consultation with three key stakeholders.

**Results:**

The study screened 1,191 publications, selecting fifty-eight (*n* = 58; *n* = 52 observational and *n* = 6 intervention) studies. Findings revealed that resilience in this context is closely linked to more than forty factors across four levels (individual; family; school/peers; and community). Pivotal factors include social and emotional competence, optimism, and family/peer relationships. While existing interventions targeting resilience show promising results, few programs are available and generalizable to different NDDs. Stakeholders highlighted the importance of addressing resilience factors that are not targeted in existing interventions: caregivers' self-efficacy and self-esteem, as well as youth's and caregiver's confidence. Preferences for and advantages of online delivery for support programs and individual/group features also emerged.

**Conclusion:**

The review emphasizes the need for a holistic approach to support youth with NDDs and their families during adolescence transition. To enhance their resilience, recognizing caregivers' roles, customizing interventions, and exploring new implementation formats are avenues that align with the current evidence and opportunities for practical development in this field.

## Introduction

Neurodevelopmental disabilities (NDDs) are highly prevalent and found to affect about 1 in 6 children (1), with an estimated total of 240 million children worldwide (2). NDDs, such as cerebral palsy or autism spectrum disorder (ASD) are chronic health conditions that may present life-long challenges. Children with NDDs can experience barriers in their physical, behavioral, verbal, cognitive, and social developmental trajectories, which can severely impact their participation in functional activities, leisure, and productivity (3, 4). In addition to these limitations, it is well established that children with NDDs are at a higher risk of experiencing mental health challenges than their typically developing peers (5–8), where a higher incidence (30%–50% vs. 8%–18%) of mental health disorders is reported (9). Mental health and well-being concerns are increasingly significant during life-transition periods, such as the shift from childhood to adolescence (10, 11) where many mental health disorders are being detected for the first time (12) and are known to persist into adulthood resulting in chronic and significant effects on health and social factors (13). Adolescence is a pivotal phase in life given the multitude of changes that are taking place simultaneously (e.g., physical/hormonal developments, social relationships, and new environments) (14, 15). Moreover, this transition period can be equally stressful for caregivers, who may also struggle with their mental health, yet are under pressure to rapidly adjust to support their child through their hardships (16).

In the context of adolescent transition, over the past decade, there has been a notable surge in anxiety and depression rates among the general youth population (17). This trend has been attributed, in part, to a decrease in independent engagement opportunities among youngsters (17), which are known to promote self-regulation (18). The importance of self-regulation cannot be overstated, as it contributes to the acquisition of skills crucial for coping with stressors and navigating vulnerable periods in life (19–23). In addition, emerging research highlighted the role of technology and social media, accessed by a staggering 97% of teenagers (24), in exacerbating mental health concerns such as anxiety, depression, and low self-esteem. The detrimental effects of excessive social media use extend further, with adolescents reporting increased incidents of cyberbullying and technology addiction (25). Notably, there exists a direct correlation between adolescents' social media usage levels and subsequent risks of self-harm (26). A survey conducted by the *Youth Risk Behavior Surveillance System* underscores the gravity of the situation, revealing that nearly 20% of high school students in the United States have seriously contemplated suicide, shedding light on the pressing adolescent mental health crisis (27). Considering the pervasive mental health struggles experienced by neurotypical children and adolescents, it is unsurprising that those with NDDs find themselves increasingly vulnerable to these challenges.

Resilience, defined as the ability to overcome life challenges and encompassing protective and vulnerability factors, becomes a crucial aspect during this transitional phase (28). Individuals use internal and external resources (protective factors) to surmount vulnerability factors. Protective factors refer to skills, strengths or physical resources that support individuals' ability to manage health conditions and strengthen their ability to overcome adversity. For instance, problem-solving abilities, emotional health, and community support may greatly impact overall levels of resilience. Conversely, vulnerability factors are elements that contribute to the worsening of health conditions. For instance, vulnerability factors may include bullying, lack of familial support, social isolation, and communication impairments (14). When vulnerability factors outweigh protective factors, overall well-being is likely to decline, and there is an increased risk of developing health conditions that could negatively impact critical life transitions.

Resilience during the shift from childhood to adolescence appears to be a particularly powerful tool for adolescents with NDDs and their caregivers when navigating this distinct time (29). The adolescent phase is known to be marked by an increased inclination towards risky behaviors, jeopardizing their health and well-being. This vulnerability extends to mental health challenges, encompassing depression, suicidal behaviors, eating disorders, and substance abuse. The positive or negative progression of adolescents' development hinges on the risks and protective factors they encounter. The dynamic interaction between these factors plays a crucial role in shaping resilience mechanisms (30). Consequently, it was suggested that research emphasis should be placed on identifying factors contributing to adolescent resilience (29, 31). While a recent systematic review provided a comprehensive overview of resilience in the general adolescent population and those with adverse experiences (31) there remains a notable gap in understanding the specific resilience factors at play for youth with NDDs as they transition into adolescence. Conducting a knowledge synthesis exercise in this area would offer valuable insights into the unique challenges and factors that contribute to resilience in adolescents with NDDs during this crucial developmental period. This would not only enhance our understanding of the nuanced interplay between resilience, NDDs, and the challenges of adolescence but also provide a foundation for developing targeted interventions and support strategies tailored to the specific needs of this vulnerable population, along with potential policy changes.

The purpose of this scoping review was to explore the concept of adolescent resilience in youth with NDDs and their families. Specific objectives included to (1) Describe impactful resilience factors in adolescents with NDDs and their families, (2) Describe how resilience is addressed as part of targeted interventions, and (3) Identify existing gaps in this field's research and clinical practice.

## Methods

### Study design

The Arksey and O'Malley framework (32), later expanded on by Levac et al. (33) was used to guide the methodology of this review in six stages.

#### Step 1—Identify the research question

The research question of this scoping review is:

*What resilience factors are impactful in adolescents with NDDs and their families, and how is resilience addressed as part of targeted interventions for this population?*

#### Step 2—Identify relevant studies

We conducted a comprehensive literature search in the following databases (*n* = 5): Ovid MEDLINE(R) and In-Process, In-Data-Review and Other Non-Indexed Citations 1996 to February 27, 2023, Social Work Abstracts 1968 to December 2022, Embase 1996 to 2023 Week 08, PsycINFO 2002 to February Week 3 2023, PubMed NCBI National Library of Medicine. The search was performed on February 28th, 2023, and embedded three main themes, including resilience, adolescence, and neurodevelopmental disabilities (Supplementary Material S1). All study designs were considered (e.g., randomized clinical trial, observational design) if they focused on the concept of resilience (or its individual/family/peers-school/community related factors) in adolescents with NDDs (mean age between 10 and 18 years old) and their families (e.g., caregivers and/or siblings). No date limit was applied. No language limits were applied. Unpublished or grey literature was excluded because we aimed to examine existing evidence-supported approaches. When information about important resilience factors or resilience interventions could not have been extracted from the publication (e.g., the design of a measure, a short conference abstract), the citation was excluded.

#### Step 3—Select studies

Citations found using the search strategy were exported and de-duplicated using reference software (EndNote^TM^ 21). Following scoping review guidelines, authors TO and NZ individually and independently proceeded to the selection by title and abstracts. All the identified citations by abstract and title were then assessed for full-text eligibility by NZ following training from the senior author (TO). Uncertainties were resolved through discussion between NZ and TO. Once the studies were selected, the reference lists were searched manually to see if any additional records met the inclusion criteria.

#### Step 4—Chart the data

Two extraction forms (one for observational and one for intervention studies) were developed *a priori* by senior author (TO) and revised by co-authors for completeness and relevance. Extraction forms included the citation details [author(s), year, country], study design and objective, definition of resilience, theories/models used, sample size and description of sample, outcomes/measurement, and study findings. For intervention studies, we extracted information on the intervention content, duration, frequency, participants, and delivery methods. For both types of studies, the resilience factors that were addressed were clearly identified. Following the development of the extraction form, two eligible articles were included in a pilot extraction phase by the senior author (TO). Another author (NZ) was trained in data extraction using the forms by the senior author and completed the extractions on all remaining citations. The senior author (TO) verified 100% of all extracted data and resolved any remaining inconsistencies or uncertainties.

#### Step 5—Collate, summarize, and report the results

A coding scheme (Supplementary Material S2) was developed by the senior author (TO) based on recent and comprehensive knowledge synthesis and consensus projects related to child and adolescent mental health and resilience-focused interventions (28, 34–36). The coding scheme includes four main resilience levels. These are individual (internal protective factors) as well as family, school/peers, and community levels (external protective factors). When extracting data from the selected publications, the coding scheme was applied to each citation to describe which factors were addressed in the intervention and the corresponding assessment and descriptive studies.

When possible, a numerical summary analysis was used to describe the study characteristics, methodology, and outcomes. An inductive content analysis was used to summarize additional information that could not be quantified (37). Once this was complete, all authors reviewed the results to ensure consistency and validity.

#### Step 6—Consultation exercise involving key stakeholders

Engaging stakeholders in the discussion on scoping review findings can validate findings, promote understanding of results, and identify important gaps (19). We recruited a key stakeholder advisory committee composed of two caregivers (*n* = 1 mother, Mrs. L., of a 10-year-old boy with CP [i.e., approaching adolescence]; *n* = 1 mother, Mrs. N., of two young 15 and 22 years old boys with ASD [i.e., passed adolescence transition]) and one young adult, Mr. M., a 28 years old man with CP [i.e., passed adolescence transition].

Individual, one-time, semi-structured online consultation meetings (45–60 min in duration) were conducted with the stakeholders to support the interpretation of study results and discuss their perspectives. To begin, the results of the scoping review were presented to participants in the form of a short PowerPoint presentation. This included an outline of the most salient resilience factors that were identified, as well as a description of available intervention programs. The discussion included the following questions, prompting participants to reflect on these factors and on their own experience (the varied questions reflect the diverse life experiences/different life stages of the participating stakeholders):

(1) For Mr. M. and Mrs. N.: Think about your/your child's teenage years, what was most difficult to overcome and why? What was helpful and how? What would you have liked to get as support back in those days?
(2) For Mrs. L.: What are your biggest concerns with regards to your child approaching the teenage years? What do you think would be helpful to be included in a coaching intervention for caregivers and children to facilitate this transition?
(3) For all: What do you think about the delivery methods (e.g., group vs. individual, online vs. in-person) of a resilience-coaching intervention?

## Results

**Figure 1:**
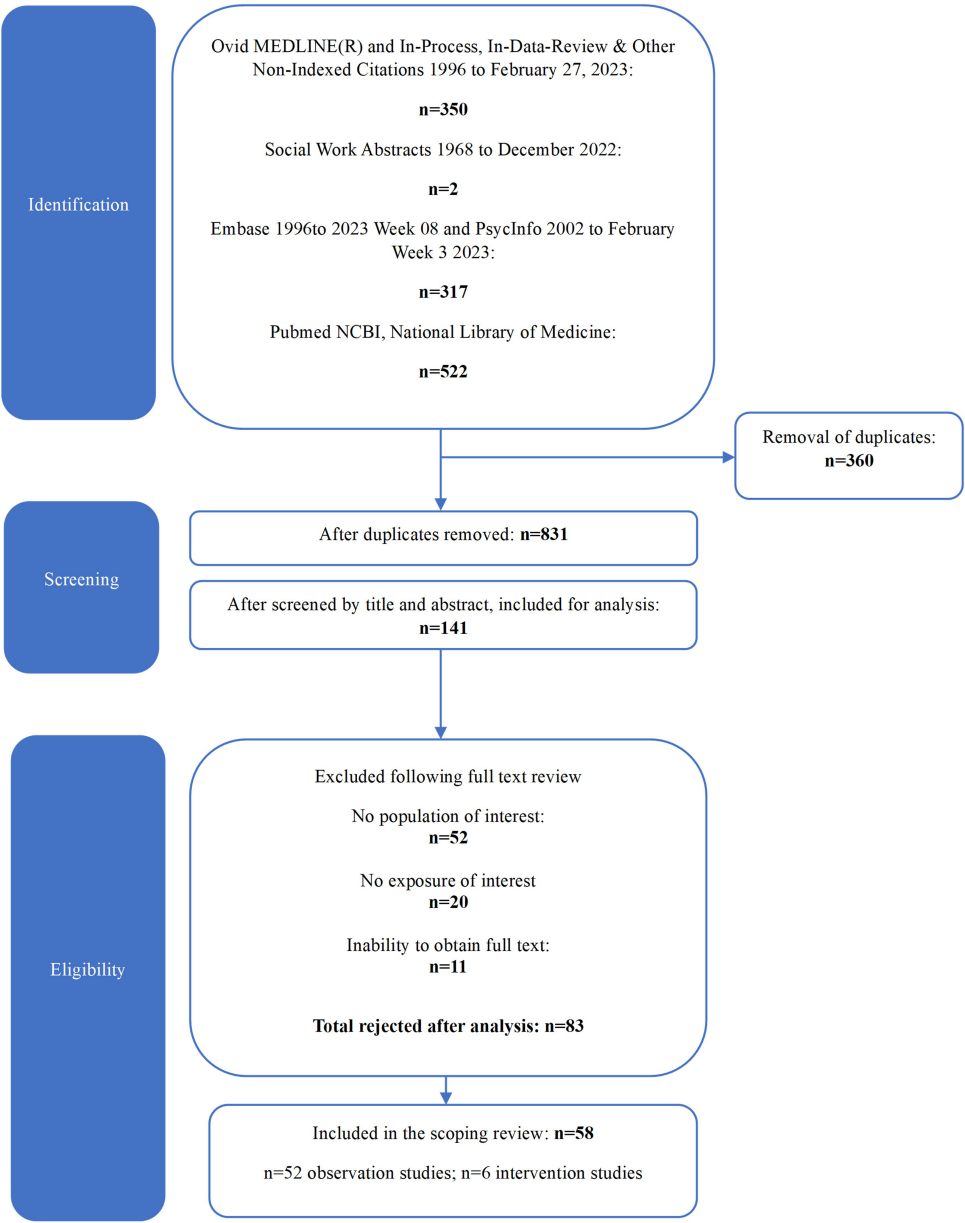
PRISMA flow chart.

The study selection process is outlined in Figure 1. Our search revealed a total of 1,191 citations. Following duplicates removal, screening by title and abstract and full text, a total of 58 publications met our inclusion criteria and were included for analysis. Of these, 89.6% (*n* = 52 studies) were observational studies and 10.3% (*n* = 6) were intervention studies. The main reasons for excluding citations during the full text review were related to the population (e.g., mean age outside of 10–18 years old range and/or no presence of NDDs, *n* = 52, 62.6% of excluded citations) and exposure (i.e., work unrelated to the concept of resilience or measurement tool development, *n* = 20, 24.1%). A full list of excluded citations with reasons is available in Supplementary Material S3.

Tables 1, 2 provide an overview of all included studies. Over 60% of the observational studies were published after 2015 and in North America. These were mainly mixed-method, cross-sectional, and longitudinal study designs. In more than 30% of these studies, the sample size was greater than 200 individuals. The main population groups that were addressed were adolescents (and/or their caregivers and siblings) with ASD (*n* = 21, 40.4% of observational studies) and those with attention deficit and hyperactivity disorder (ADHD, *n* = 11, 21.1%). Resilience was defined in most assessment studies (*n* = 32, 61.54%) as a dynamic coping adaptation in the context of adversity and despite challenging circumstances, and many projects were anchored in a resilience-related framework or model (*n* = 34, 65.3%) (e.g., Social Ecology of Resilience Theory, Resiliency Models of Family Adjustment and Adaptation) (Supplementary Material S4A).

**Table 1:** Characteristics of included intervention and observation studies.

Characteristics of included observational studies	Number of studies (*n*)	Percentage of assessment studies (%)
Year published		
≤2010	6	11.5
2011–2014	12	23.0
2015–2019	15	28.8
2020–2023	19	36.5
Geographic region		
North America	32	61.5
Europe	11	21.1
Asia	2	3.8
Australia	3	5.7
South Africa	2	3.8
South America	2	3.8
Study design		
Mixed-method studies	6	11.5
Cross-sectional studies	24	46.1
Longitudinal/cohort studies	10	19.2
Case studies	4	7.6
Qualitative studies	5	9.6
Other	3	5.7
Diagnostic group		
Autism spectrum disorder	21	40.3
Attention deficit and hyperactivity disorder	11	21.1
Developmental disabilities (other, e.g., developmental coordination disorder, cerebral palsy)	8	15.3
Intellectual disabilities	7	13.4
Traumatic brain injury	5	9.6
Theoretical mode/framework		
Walsh theory of family resilience	3	5.7
Resiliency models of family adjustment and adaptation (RMF; McCubbin)	3	5.7
Grounded theory approach	2	3.8
Social ecological/ecocultural theories (social ecology of resilience theory; SERT)	5	9.6
Risk-resilience models	3	5.7
Stress buffering, direct and psychopathological models	2	3.8
Family systems/adaptation theory	2	3.8
Other noted theories/frameworks	14	26.9
Total sample size		
<20	5	9.6
20–50	11	21.1
50–100	10	19.2
100–150	5	9.6
150–200	3	5.7
>200	17	32.6
Undefined	1	1.9
Characteristics of included intervention studies	Number of studies (*n*)	Percentage of intervention studies (%)
Year published		
≤2017	2	33.3
≥2018	4	66.6
Geographic region		
North America	3	50
Australia	3	50
Study design		
Mixed-method studies	2	33.3
Randomized control trials	4	66.6
Diagnostic group		
Autism spectrum disorder	3	50
Developmental disabilities (other, e.g., developmental coordination disorder)	2	33.3
Attention deficit and hyperactivity disorder	1	16.6
Resilience framework	1	16.6
Integrated autism (autism CRC) conceptual model	1	16.6
Index for inclusion framework	1	16.6
Positive psychiatry model	1	16.6
Universal coping program	1	16.6
Universal mental health literacy framework	1	16.6
None	1	16.6
Total sample size		
<20	1	16.6
20–50	2	33.3
50–100	1	16.6
>100	2	33.3

**Table 2:** The characteristics and results of included intervention studies per population group.

Reference	Year	Country	Study design	Population	Resilience related intervention	Resilience related assessment(s)	Results
				• Target group • Sample size (*n*) • Age (range; mean ± sd) • Gender (M|F %)	• Construct(s) addressed: Coding • Duration/frequency • Delivery mode • Receiving client(s) • Provider(s)/professional discipline	• Tool: Construct(s) measured	(+) significant/ (-) nonsignificant between-group differences (RCTs) OR (+) significant improvements/ (-) nonsignificant improvements pre-post (non-RCTs) Summary of Qualitative Findings
Autism spectrum disorder (ASD)							
Mackay et al. (1)	2017	Australia	RCT	• Adolescents with ASD in years 6 and 7 of school • n = 29 • 10–13 yrs old; 11.8 ± 0.7 yrs old • 90|10	• Cognitive Behavioral Therapy components including stress management using self-management and relaxation strategies, positive self-talk, problem solving strategies, solution-focused. • Interpersonal Psychotherapy components, including promoting connectedness with support network, develop skills to reduce interpersonal conflict, promote harmony with self and others. • A3, 17, 22, 24; B1, C1; D2 • 50 min × session, 1 session × week × 11 weeks (11 learning modules) • In person, individually, in school setting, manualized • Adolescents with ASD • Trained facilitator—coach/psychologist	• Child Depression Inventory (CDI): Severity of depression symptoms. • The Coping Self-Efficacy Scale (CSES): Individual's confidence to respond adaptively to stressful events. • Strengths and Difficulties Questionnaire (SDQ): Behavioral and emotional mental health issues.	At post-treatment (11 weeks): (-) CDI (self-report) (-) CSES (self-report) (-) SDQ (self-report) (+) CSES (parent-report) (-) SDQ (parent-report) (-) SDQ (teacher-report) Qualitative results indicate perceived improvements from the intervention on adolescents’ coping self-efficacy, self-confidence, social skills, and affect regulation.
Shochet et al. (2)	2022	Australia	Longitudinal, mixed methods	• Adolescents with ASD in years 7 and 8 of school • n = 30 • 11–14 yrs old; 11.84 ± 0.86 yrs old • 80|20	• Integrated model accounts for RAP-A, RAP-P and RAP-T • Resourceful Adolescent Program for Adolescents • The intervention, consisting of the adolescent, parent and teacher components of the Resourceful Adolescent Program–Autism Spectrum Disorder (RAP-ASD) augmented with the Index for Inclusion, was implemented. **Based on this model, a multilevel, selective, evidence-based resilience intervention was designed to address the reciprocally related protective factors of self and affect regulation and school connectedness to improve the wellbeing of adolescents on the spectrum. • RAP-A: A2, 3, 4, 7, 17, 21, 22, 23, 26, 28; C1 • RAP-A was implemented over 2 years in the second and third terms of the Australian school year (April-September 2016 and 2017) • 50 min × session, 1 session × week × 11–14 weeks (11-session group program delivered weekly across one school term but can also be delivered in a selected or indicated format) • In person, one-on-one during a non-core lesson in the school day in a room separate to the classroom • Adolescents with ASD • Trained facilitators • Resourceful Adolescent Program for Parents • RAP-P is a strength-based, non-blaming resilience-building program. It is based on an integration of cognitive-behavioural theory (CBT), Bowen Family Systems Theory (Kerr & Bowen, 1988; Titelman, 2014), and knowledge from developmental psychology of the maturational changes that occur naturally during adolescence. • RAP-P*: A4, 17, 20, 25, 26; B1, B2, B4; D1 • RAP-P was implemented over 2 years in the second and third terms of the Australian school year (April-September 2016 and 2017). Facilitators delivered RAP-P-ASD by following the session content and process described in the RAP-P-ASD Group Leader's Manual (Shochet & Wurfl, 2016a), and each parent participant received a RAP-P-ASD Participant Workbook • 2 h workshops, 1 session × week × 4 weeks (separate workshops conducted for each participating school) • Parents of adolescents with ASD • Trained facilitators	• The Coping Self-Efficacy Scale (CSES): Increased coping self-efficacy • Strengths and Difficulties Questionnaire (SDQ): Improved behavioural and emotional functioning • Children's Depression Inventory 2 (CDI 2): reduced depressive symptoms • Self-report Psychological Sense of School Membership Scale (PSSM): school belonging, in the form of school connectedness (extent to which a student feels accepted, valued, and supported in their school environment) • Anxiety Scale for Children with Autism Spectrum Disorder (ASC-ASD): anxiety	• Resourceful Adolescent Program for Adolescents Statistically Reliable Change: At 3 months follow-up (+) ASC-ASD (+) SDQ-TD (+) PSSM (+) CSES (+) CDI 2;(with particularly high percentages of improvement reported at 3-month follow-up) At 12 months follow-up (+) PSSM (+) CSES • Resourceful Adolescent Program for Parents Statistically Reliable Change (+: stable improvement across all time points) coping self-efficacy Peak at 12-month follow-up (+) CDI 2 (parent-report) (+) ASC-ASD (parent-report) (+) PSSM (parent-report) Clinically Significant Change (mainly at 6-month follow-up) (+) CDI 2 (parent-report) (+) ASC-ASD (parent-report) (+) SDQ-TD (parent-report)
Kuhlthau et al. (3)	2022	USA	Randomized pilot trial	• Adolescents with ASD and their NT siblings Adolescents with ASD • n = 45 • Age of adolescents with ASD: 12–16 yrs old; (46.7% of participants with ASD or 21/45) • No information provided on gender of individuals with ASD NT Siblings • n = 40 • Age of NT siblings: 14–17 yrs old; (M = 15; SD = 1.2) • Gender of NT siblings: • 49|51	• Modified Stress Management and Resiliency Training-Relaxation Response Resiliency Program (Modified SMART-3RP, known as SibChat) • The SMART-3RP, includes three core skills-training components: mind-body practices, cognitive-behavioral therapy (CBT) skills, and positive psychology approaches (Specific skills included: single pointed focus, body scan, mindful awareness, yoga, insight imagery, contemplation, loving kindness, and idealized self.) *Modified SMART-3RP was referred to as SibChat and was built upon 2 earlier versions of the SMART-3RP. • Siblings: A4, 12, 16, 18, 20, 22, 23, 24, 25, 28; B1, • 60 min × session, 1 session × week × 8 weeks (8 sessions)—Intervention sessions ran from May to November 2020 • The first cohort started in May 2020 and the second in July 2020. • Virtual, group conference sessions • SibChat was delivered using a HIPAA compliant online video conferencing platform. Participants received a workbook and audio recordings of the RR exercises weekly. • NT siblings (aged 14–17) of adolescents with ASD (primarily aged 12–16) • A clinical psychologist and clinical psychology postdoctoral fellow trained to implement SibChat conducted the intervention and participated in weekly clinical supervision to assure quality and adherence to the intervention model. The study team also met weekly to discuss and resolve any issues that arose in the groups.	• Current Experiences Scale (CES) and the Measure of Current Status-A (MOCS-A): resiliency (adapted from the Post Traumatic Growth Inventory) • Penn State Worry Questionaire (PSWQ): worry (about the frequency, duration and persistence of worry) • 0 to 10 analog scale = Distress • 2 PROMIS (Patient Reported Outcomes Measurement Information System) self-report measures (developed for youth aged 8 to 17, the v2 short forms): depressive symptoms and anxiety symptoms • 1 item from the PROMIS sleep disturbance scale: sleep disturbance • The positive affect subscale of the Positive and Negative Affect Schedule for Children (PANAS-PosC): positive emotions • The Child and Adolescent Mindfulness Measure (CAMM): trait mindfulness	At 3 months post-baseline: (+) Measure of Current Status-A (+) Current Experiences Scale (+) PSWQ (+) CAMM (-) PROMIS-D (-) [PANAS-C (PA)] (-) PROMIS-S
Other developmental disabilities							
Ahmadi (4)	2020	USA	RCT (clinical trial, randomized with parallel assignment)	• Adolescents with ADHD & PTSD • n = 11 • 10–15 yrs old; 11 ± 3 yrs old • 45|55	• Reminder Focused Positive Psychiatry (RFPP) (8/11 participants) RFPP intervention was inclusive of positive psychiatry interventions on (1) traumatic reminders and (2) avoidance and negative cognition. • A2, 3, 4, 7, 11, 12, 16, 17, 19, 22, 24, 25, 27, 28; B1, 2; C1; D1 • 75 min × session × 2 sessions × week × 6 weeks (12 sessions)—Intervention sessions ran from September 2016 to June 2018. • In person, Group therapy • Adolescents with ADHD and PTSD • 2 therapists trained in positive psychiatry • Trauma-focused CBT (TF-CBT) (4/11 participants) The trauma narrative and processing components enabled the child to talk about memories individually and in groups. The final sessions focused on grief-specific elements. The structure of all sessions were as follows: (1) refreshments, (2) review the previous group activities, (3) teach new components, (4) assign homework, and (5) preview the next group. • A2, 3, 4, 12, 16, 17; B2, C1; D1 • 2 sessions × week × 6 weeks (12 sessions)—Intervention sessions ran from September 2016 to June 2018. • In person, Group therapy • Adolescents with ADHD and PTSD • 2 therapists trained in TF-CBT	• ADHD Swanson, Nolan, and Pelham (SNAP) Questionnaire: perceived health status • PERMA: positive emotion, engagement, relationships, meaning, and accomplishment • Gratitude Resentment and Appreciation Test (GRAT): gratitude, resentment and appreciation • UCLA Trauma Inventory: traumatic reminders • Posttraumatic Growth Inventory (PTGI): positive changes after experiencing PTEs in the 5 domains of Relating to Others, New Opportunities, Personal Strength, Spiritual Change, and Appreciation of Life. • Connor-Davidson Resilience Scale: psychological resilience in patients with posttraumatic stress disorder (PTSD) and other psychiatric ailments psychological resilience in patients with posttraumatic stress disorder (PTSD) and other psychiatric ailments • Clinician-Administered PTSD Scale for DSM-5-Child/Adolescent Version (CAPS-CA),: PTSD scale based upon DSM-5 criteria	At 6 weeks: For RFPP group: (+) PERMA (+) GRAT (+) PTGI (+) CAPS-CA (decrease) (+) SNAP (decrease) For TF-CBT group: (+) CAPS-CA (+) SNAP At 12 months: For RFPP group: (+) PERMA (+) GRAT (+) PTGI (+) CAPS-CA (+) SNAP For TF-CBT group: (+) CAPS-CA (+) SNAP
Firth (5)	2013	Australia	Mixed methods clinical trial	• Adolescents with and without speech language disability (SLD)/ dyslexia (post-screening, last year of primary school) • n = 102 • 10–11 yrs old; 10.6 • 45|55 • “Contrast Group”: Adolescents with and without SLD/ dyslexia (post-screening) • n = 39 • 10–11 yrs old; 10.6 • 41|59	• Universal classroom coping program: • This program was based on cognitive behavioural therapy principles and involved awareness of current coping strategies, use of positive thinking, assertion, goal setting and problem solving • A4, 11, 12, 15, 16, 17, 19, 20, 24, 28; C1, C2, C3, C4, C5; D1 • 50 min × session × for 10–11 weeks (10 sessions) • In person, group delivery • All year 6 students, including those with and without a diagnosis of dyslexia • Assistant principal or leading teacher • Withdrawal dyslexia coping program • Included role modelling by successful adults who have dyslexia. It also included direct teaching of the efficacy of taking control in the face of dyslexia, development of individual awareness of current coping approaches to having dyslexia, opportunities for individual investigation of dyslexia, discussion of dyslexia-related issues in a supportive group and individualized support for a dyslexia-related goal. • A1, 3, 4, 19, 20, 22, 23 • 10 additional sessions (minimum) concurrent/withdrawal sessions held for students with dyslexia • In person, group delivery • Students with dyslexia • Adults with dyslexia Note: school staff also received training as part of this program; addressing the following components: C1, 2, 3, 4, 5; D1, 2, 3, 4, 5	• The Locus of Control Scale for Children (LCS): distinction between perceived control over life (internal locus of control) and perceived control by external circumstances (external locus of control) • Arc Determination Scale (ADS): self-determination or feeling of being in control • Adolescent Coping Scale (ACS): coping (with particular concerns; productive vs. non-productive coping strategies) • Reynolds Adolescent Adjustment Screening inventory (RAASI): well-being • Victoria Social Questionnaire for Secondary Students (VSQSS): school engagement	Primary School Intervention Group: Comparison Between Students with and without Dyslexia Post-Intervention (t1): (+) ACS (within group) (-) ACS (between) (-) VSQSS At 1 Year Follow-up (t2): (+) ACS (within group) (-) ACS (between) (-) VSQSS Transition to Secondary School: Comparison of Intervention and Contrast Group Participants At 1 Year Follow-up (t2): (+) RAASI (contrast group scoring higher I.e. less well-being, than intervention group) (+) ADS (+) ACS (intervention group with SLD/dyslexia showed an increase in ADS and ACS in comparison to the control group with SLD/dyslexia)\
Katz et al. (6)	2020	Canada	Cluster RCT	• Adolescents with developmental disabilities (ASD, ID, fetal alcohol spectrum disorders) in grades 3–12 • n = 113 total Intervention • 9–18 years old • Mean grade level = 7.64 (ages 12–13) • 56|44 Wait list control • 9–18 years old • Mean grade level = 7.23 (ages 12–13) • 60|40	• Universal mental health program (Universal SBMH) combining dialectical behavior therapy (DBT): elements of CBT and mindfulness, including skill modules, group therapy and one to one therapy + mental health literacy (MHL): involves developing knowledge related to mental health skills for managing one's own well-being, recognizing when oneself or another is struggling etc. (p. 4071) • DBT Skills were taught through 4 adapted modules with differentiating activities based on age, communication skills, and learning modalities. 3 sessions per module × 4 modules = 12 sessions (M1: Interpersonal effectiveness skills, M2: Emotional regulation, M3: Mindfulness, M4: Distress tolerance) • MHL Program used was called “The Brain Unit” and was designed to connect to curicculum, SEL and DBT. 9 detailed lessons were given to teachers (L1-4: neuroanatomy focus to gain understanding of brain structures, function and the role of neurons in neurochemistry, L5-6: exploration of the cortisol cycle, biological responses related to stress and the role of cognitive triggers in emotional responses, L6: role of 5 sense in emotional memory, L7: mental health and emotional literacy, L8: mental illness continuum and connections between physical and mental illnesses, L9: issues of stigma and media portrayal) • A2, 3, 4, 7, 8, 10, 12, 16, 17, 19, 20, 21, 22, 23, 24, 25, 26, 27, 28; C1, 3, 4; D2, 5 • The nine lessons of the MHL program and four modules of the DBT skills were separate lessons and were implemented sequentially. • In person, group/school setting (intervention implemented class wide as part of the schools’ health curriculum) • Adolescents with and without developmental disabilities (DD) • Classroom teachers	• Self-Description Questionnaire–General Subscale (SDQ): Self-concept • Resilience Inventory: Self-efficacy subscale (RI-9): Coping skills, management of challenging situations • Global Portrait of Social and Moral Health for Youth (GPSMHY): Social support	After 3 months: Between baseline and mid-program (October to January) Control vs. Intervention (+) SDQ (self-report) (+) RI-9 (self-report) (+) GPSMHY (self-report) (+) fidelity to program (teacher-report)

**Figure 2:**
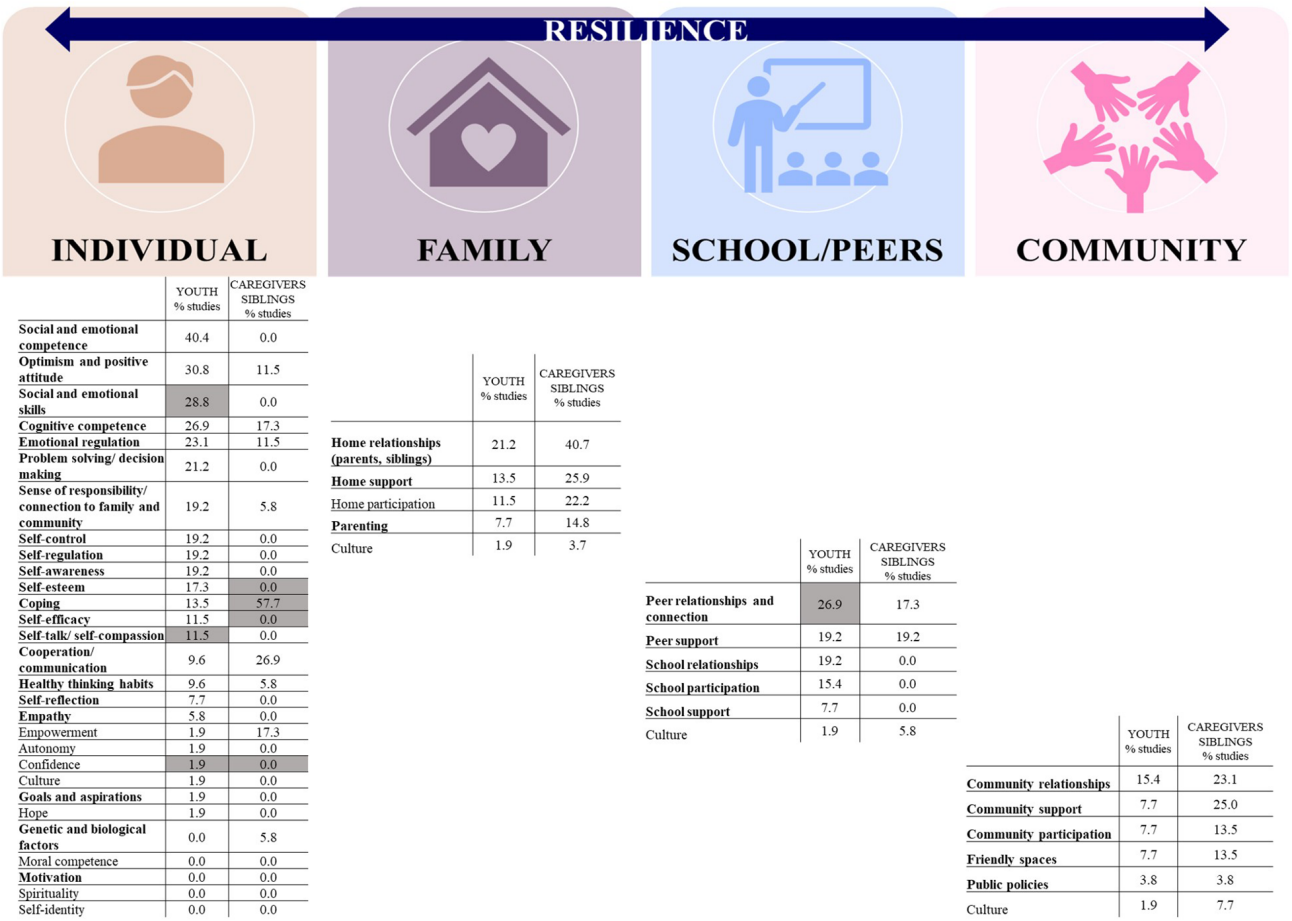
Listed factors with percentages refer to significant resilience factors in assessment studies (% of studies reporting these factors). Bolded factors refer to resilience factors that were addressed as part of interventions in intervention studies highlighted factors (in gray) refer to resilience factors identified as important through discussion with key stakeholders.

The coded resilience factors that were addressed as part of these studies at large and emerged as significant are displayed in Supplementary Material S5. Figure 2 outlines the main resilience factors that emerged from observational studies. It includes factors that were found to be significantly associated with the individuals' resilience levels and/or as having a mediating effect in regression models defining resilience. For an adolescent with a disability, the most salient *individual-based* resilience factors were social and emotional competence (40.4% of studies), optimism and positive attitude (30.8%), social and emotional skills (28.8%), cognitive competence (26.9%), and emotional regulation (23.1%). For caregivers and/or siblings, impactful *individual-based* resilience factors were coping (57.7%), communicational and cooperation (26.9%), and empowerment (17.3%). For both groups, in terms of *family, school/peers, and community-based* resilience factors, home relationships (21.2% of studies—adolescents; 40.7%—family), peer relationships and connections (26.9%; 17.3%), as well as community relationships (15.4%; 23.1%) were respectively identified as critical.

All intervention studies (*n* = 6/6, 100%) were published after 2017, where 50% were completed in North America and 50% in Australia. Most (*n* = 5/6, 83.3%) were randomized clinical trials. In intervention studies, the concept of resilience was defined in four studies and five studies mentioned the use of a resilience-related framework or model (e.g., Integrated Autism Conceptual Model, Index for Inclusion Framework) (Supplementary Material S4B). Over 50% of intervention studies had a sample size of more than 50 participants. The main population groups that were addressed in these interventions were adolescents (and/or their caregivers and siblings) with ASD (*n* = 3 studies, 50% of intervention studies), learning disability and a mixed of developmental disabilities (*n* = 2, 33.3%), as well as ADHD (*n* = 1, 16.6%), with mean age of 12.1 ± 1.5 years old (Table 1). On average, 10.8 ± 1.5 intervention sessions were offered, and most were in-person (*n* = 5/6, 83.3% of studies). All studies provided individual interventions, while some (*n* = 3/6, 50%) offered a group component. Interventions were mostly delivered by trained specialists/coaches, psychologists, or teachers. Overall, the existing interventions were found to be effective in improving resilience in general, including resilience-related components such as coping mechanisms, self-efficacy, positive emotions, behavioral and emotional functioning, and sense of connectedness or belonging (Table 2). Figure 2 further depicts the resilience factors addressed as part of these interventions (bolded). We found that multiple *individual-based* factors of resilience (e.g., self-regulation and communication) were addressed in most projects targeting youth. However, *caregivers' individual-based* factors of resilience (problem solving, decision making, self-efficacy, self-reflection, and sense of responsibility) were addressed in only one study. For siblings, individual factors such as coping and positive thinking were targeted in one intervention. In *school/peers and community-based* factors, peer relationship and connectedness were commonly addressed (66.7% of studies), along with community relationships and support and friendly spaces (50% of studies). It is important to note that several *individual-based* resilience factors (e.g., confidence and autonomy) were not addressed as part of interventions yet they have emerged as important in mediating one's resilience.

Semi-structured interviews with key stakeholders revealed the importance of additional factors. Mrs. L. reported that her main concern regarding her son revolves around his transition to high school and his ability to build and sustain peer relationships. She refers to an *individual-based* factor of self-advocacy, a skill that she wishes to instill in her son.

Mrs. N. reports that the caregiver's internal assets, such as coping, confidence, and self-esteem, are essential:

*“Resilience is a skill that is built over time and through experience. So, coping and having good and healthy coping mechanisms is critically important, no question.”*

*“Having a neurodiverse child impact directly our level of confidence, our level of competence, our level of self-esteem in terms of that role of now being a parent of a child with special needs.”*

For Mr. M., the individual factors of negative thoughts and self-talk were impactful when going through adolescent transition:

*“I would have negative thoughts that would creep up where it*’*s a little bit frustrating when I can*’*t perform as well as I would like to”*.

In addition, he reports that one's confidence could be affected and in turn negatively impact resilience:

*“Confidence is something that I am always trying to improve. It was never really that high because I was always comparing myself to others”*.

This discussion also revealed that future resilience interventions to support adolescents could be delivered in an online format to enhance accessibility and feasibility. Stakeholders also reported that group sessions (in addition to individual sessions) might be beneficial:

Mrs. L.: “*Nobody understands you quite like somebody else going through it”*.

Mr. M.: “*I think there is a lot of benefit in seeing other youth who are in similar situations. The shared experience allows you to learn some techniques that they are using and vice-versa”*.

Figure 2 further highlights resilience factors that were reported to be important by key stakeholders (in grey). For the *individual-based* resilience factor (confidence) we note that although it was identified as critical in the literature (for youth, in 1.9% of studies) and by key stakeholders (for both youth and caregivers), it is not addressed as part of existing interventions.

Other important gaps were identified. Namely, the *individual-based* resilience factors related to self-identify, spirituality, and moral competence were not examined in any of the observational studies, nor addressed as part of the existing intervention programs.

## Discussion

This review sought to synthesize evidence from a wide range of sources to describe influential resilience factors in youth with NDDs and their families as they navigate the transition to adolescence. The review also explored how resilience is addressed as part of targeted interventions and highlighted existing gaps in research and clinical practice. Our findings demonstrated that over the past decade, the concept of resilience among youth with NDDs and their families as they transition into adolescence has been identified as an important topic to understand and develop. A growing body of evidence illustrates that fostering resilience in youth with NDDs and their families is a multifaceted process, with nearly forty emerging essential factors.

### Adolescent perspectives

Our findings pinpointed several *individual-based* protective factors in youth with NDDs that arose from more than 20% of selected observational studies. These include emotional and social competence, positive attitude, emotional regulation, as well as problem-solving and decision-making.

Emotional and social competencies refer to the adolescents' ability to successfully manage their emotional arousal and positively engage in social settings (38). Adolescence is a period of significant growth in which social and emotional development shapes youth's trajectory (39, 40). Fostering one's emotional awareness is foundational to this process; it includes recognizing and labeling feelings, understanding the sources of emotions, and being in touch with one's strengths and weaknesses (41). A recent study examined the associations between emotional competence and prosocial behaviors with peers among children with ASD. Authors found that those with ASD showed significantly lower rates of emotion regulation and use of discrete coping strategies during peer interactions in comparison to their neurotypical peers (42). Another study conducted with neurotypical adolescents evidenced that social competence was associated with emotional intelligence and social anxiety (43). More specifically, youth who had higher emotional intelligence and lower social anxiety demonstrated overall stronger social skills. In addition, it is suggested that these skills are important for adolescents' general engagement in social activities (43). This evidence suggests that social and emotional competencies are integral to positive social relationships. Thus, we propose that skill development in this area should be considered as an integral aspect of future resilience interventions for youth with NDDs and their families.

Our scoping review also determined that emotional regulation was a key resilience factor in adolescents with NDDs, especially given that young people experience a wide range of distinct turbulent emotions (44). Emotional regulation refers to one's ability to not only understand their emotions but also to have control over which emotions are experienced, as well as when and how they are experienced and expressed. In 1994, Thompson explained that “emotion regulation consists of the extrinsic and intrinsic processes responsible for monitoring, evaluating, and modifying emotional reactions, especially their intensive and temporal features, to accomplish one's goals” p. 28–29 (41). It includes recognizing and labelling one's feelings, understanding the triggers for said emotions, and distinguishing between different emotional states (45). In fact, emotional regulation is found to be affected in children (46, 47) and adolescents (48, 49) with NDDs. Nonetheless, emotional regulation, as a resilience factor, was found to be addressed in only 50% of the selected intervention studies, and primarily in adolescents with ASD and ADHD. In relation to that, we identified an important gap in targeted resilience interventions for youth with CP. Indeed, emotional regulation is a major challenge in children with CP that commonly translates and intensifies in adolescence and significantly affects multiple life areas such as peer interactions, relationships and overall mental health (49–52). Consequently, we advocate for future developments in resilience coaching programs for children with physical developmental disabilities, such as CP.

Furthermore, youth's optimism and positive attitudes were found to play a significant role affecting their resilience and well-being. In accordance to our finding, a recent systematic review of 31 studies and a cohort of 46,262 adolescents aged between 13 and 17 years old showed that optimism contributes significantly to their overall mental health (53). The review concluded that optimism and positive attitude act as “buffers against the impact of stress, […] pathological symptoms and risky behaviors” (53). Overall, positive thinking can help adolescents better manage stress and cope with the challenges of this transitional period (54). Similarly to emotional regulation the factor of optimism and positive attitude was addressed in 50% of the intervention studies, and this primarily among youth with ASD, ADHD, and language disorders. Provided that over 30% observational studies have determined this factor to be influential (i.e., second on the list of most common significant factors) and the importance of optimism to overall youth's well-being, we suggest that future coaching interventions supporting resilience ensure its inclusion.

In addition, our review pinpointed that problem-solving and decision-making are equally important factors that contribute to resilience. In fact, youth with NDDs are particularly likely to face challenges in these areas (55, 56). Studies evaluating existing interventions have shown that improving the problem-solving and decision-making skills of teenagers with NDDs can result in improved independence, day-to-day functioning, and general health (57–60). Therefore, optimizing these capabilities has immense potential.

Our review also explored family and peer relationships as key protective *home-based* and *school-based* factors. The well-being and resilience of adolescents with developmental impairments is supported by a loving and caring family environment that offers emotional support, open communication, and a consistent schedule (61). Equally important are the experiences and connections adolescents have with peers in the school environment, as positive peer relationships may improve emotional well-being, school achievements (62, 63), psychological adjustment (64, 65), and self-esteem (66, 67). The research suggests that adolescents with NDDs benefit significantly from inclusive educational approaches because they create opportunities for social involvement and acceptance (40).

Finally, it is noteworthy that adolescents with NDDs are known to undergo a heterogeneous and complex process to develop their disability identity (68). Despite the evident importance of this factor as protective (69), our scoping review found that the concept of self-identity was not addressed as part of observational and intervention studies. Adolescents' understanding of their unique circumstances and subsequent implications is just one element that influences their path to positive self-identification, as adolescents' self-esteem may be affected (70, 71). In relation to that, our key stakeholder interviews added a qualitative dimension to this evidence, highlighting the importance of factors like self-advocacy, confidence, and self-esteem in building resilience (individually and relationally) among adolescents with NDDs. Indeed, by fostering self-advocacy through cultivating self-determination, adolescents can be empowered to actively participate in their own treatment and decision-making (72).

Moreover, the discussion with key stakeholders also revealed the potential benefits of remote interventions and group sessions for supporting adolescent transitions, as these formats can enhance accessibility and the shared experience of learning from peers. These results are aligned with previous evidence in the field of pediatric telehealth, which has been shown to be an effective alternative to traditional face-to-face methods and well accepted by caregivers and teens (73). Specifically, to address the mental health of children and youth with NDDs, a call to implement and benefit from online programs has been put forward (74).

### Caregiver perspectives

The scoping review also provided valuable insights into the experiences of caregivers who support adolescents with NDDs, highlighting the challenges and opportunities within this context. Notably, some caregivers' perspectives were intertwined with those of adolescents, as they often play a significant role in the lives of their children (61, 75). For instance, many of the included observational studies examined the various situations common during adolescence that may present issues, such as helping their children transition into high school and build and sustain peer relationships. Further, caregiver concerns are often related to the development of individual-based factors in their children, such as self-advocacy, health coping mechanisms and confidence. Interviews with caregivers revealed that their internal assets are essential in supporting their children's internal assets. These findings highlight the significance of caregivers' own well-being and mental health in promoting positive trajectories for their adolescents with developmental disabilities. The stakeholder discussion revealed that having a neurodiverse child can impact caregivers' perceived competence. Thus, more research to support caregivers in developing a positive self-concept is important as only a small number of studies addressed individual-based resilience factors in caregivers themselves. Despite this, there is great potential for positive and inclusive interventions (such as group sessions). These provide an opportunity to share experiences and connect with other caregivers ultimately to better support those with NDDs. It is indisputable that caregivers play a critical role in fostering resilience among adolescents with developmental disabilities, and the multifaceted nature of caregiving for this population cannot be overlooked.

### Common perspectives

Developing positive coping strategies emerged as a key theme for both adolescents and caregivers. This demonstrates the importance of developing adaptive mechanisms to navigate the unique challenges associated with NDDs. Self-regulation, both for individuals with NDDs and their caregivers, was similarly identified as important for encouraging healthy emotional regulation and promoting resilience. Previous research suggests that coping strategies are affected in youth with NDDs, where they often “ignore” the issues (60). In caregivers of children and youth with chronic illness, coping strategies were found to correlate with quality of life (76). Beyond employing coping strategies, young individuals with diverse NDDs have conveyed that their sense of well-being hinges on engagement and participation, interpersonal connections, family dynamics, and personal growth (77). Their perception of well-being was found to revolve around feeling supported, included, and respected, while also sensing value and capability (77). In relation to that, our review emphasized the significance of social support, clarifying the need for situation-specific resources and encouraging family environments. Nonetheless, in many studies, cultural considerations were underscored for both adolescents and caregivers. Moving forward, interventions should not only focus on coping strategies but also embrace a holistic approach that acknowledges and integrates diverse cultural backgrounds. This comprehensive approach is crucial for fostering a supportive environment that addresses the multifaceted needs of both individuals with NDDs and their caregivers.

Finally, the evidence reiterated the importance of public policies relating to adolescents with NDDs and their families. For instance, previous work in the field of leisure for children with disabilities highlighted that few policies have specific mechanisms and action plans in place (78). It emerges that there is a need for policy initiatives that not only recognize the diverse challenges faced by adolescents with NDDs and their families but also outline targeted strategies and concrete action plans. These policies should be designed to promote inclusivity, accessibility, and support across various domains, including education and health. By addressing these aspects, policymakers can contribute significantly to fostering an environment that empowers adolescents with NDDs and their families, ensuring their equitable participation and well-being in society.

### Future opportunities and limitations

The findings presented above described the state of research and interventions focusing on young people with NDDs and their families, particularly in the context of adolescent resilience. While the existing literature and interventions have made significant contributions, there remain several gaps and areas for improvement. Existing interventions often only speak to the adolescent experience, with limited attention paid to the well-being and resilience of their caregivers. Caregivers play a pivotal role in the lives of these youth and require dedicated support to effectively fulfill their caregiving responsibilities (79, 80). Future work must focus on *individual-based* factors specific to caregivers, such as self-esteem, self-efficacy, and coping, as they are closely linked to caregivers' ability to provide effective support (and, in turn, influence the resilience of their children).

Adolescent-focused interventions could further prioritize the interconnected nature of family, school, peer, and community-based factors in developing resiliency. A more comprehensive approach would address the broader context to provide a more holistic support system for youth and their families. One way to do so would be to customize interventions to cater to context-specific needs and strengths, rather than falling back on adopting a one-size-fits-all approach to health and well-being. On a similar thread, there is room for increased integration of firsthand perspectives in intervention development. Engaging caregivers and adolescents themselves in the design and evaluation of interventions will lead to more relevant, effective and sustainable solutions (81–83). Moreover, future interventions should be family-centered and address the unique challenges and strengths of both the teen and the caregiver. They should be inclusive and include the necessary tools and resources for both parties. Additionally, as suggested by stakeholders, exploring novel intervention formats (such as an online setting or a group environment) may also make support more accessible and feasible.

Our scoping review has limitations. Despite efforts to be comprehensive, it is possible that some relevant papers were overlooked, as search algorithms may not capture all potential terms used to describe resilience in this population. While a total of five databases were included, there might be a bias towards health-related literature, potentially neglecting to consider relevant studies in other domains. In addition, we conducted individual semi-structured interviews with our stakeholders. A common discussion might have resulted in additional arising themes and ideas. Moreover, we did not include experts in the field in the key stakeholder consultation exercise. Nevertheless, our team is presently launching a nation-wide survey and follow-up semi-structured interviews, exploring topics of interest for a resilience coaching program, from the perspectives of caregivers, young adults with NDDs, and experts in the field.

## Conclusion

This scoping review provided a comprehensive overview of the factors influencing resilience in youth with NDDs and their families, offering valuable insights for future research, clinical practice, and policy development in this area. These findings underscore the importance of a holistic and inclusive approach to support young people and their families throughout the complexities involved with the transition to adolescence. This type of review contributes to the ongoing dialogue surrounding adolescent resilience and offers valuable insights for stakeholders seeking to better support this vulnerable population.

The path forward in developing more comprehensive approaches and interventions to research and practice involves recognizing the indispensable role of caregivers, tailoring interventions to specific contexts, and exploring emerging implementation formats. By bridging these gaps and pursuing sustainable change, we can foster greater resilience among adolescents with developmental disabilities and create a more inclusive and supportive environment for their families. This opportunity not only aligns with the findings of the scoping review and current research landscape but also contributes to the ongoing advancement of practical development in this field.

## Data Availability

The original contributions presented in the study are included in the article/**Supplementary Materials**, further inquiries can be directed to tatiana.ogourtsova@mcgill.ca.
