# Supplementary material for: Resilience and adolescence-transition in youth with developmental disabilities and their families: a scoping review
Source: Front Rehabil Sci. 2024 Feb 27;5:1341740. doi: 10.3389/fresc.2024.1341740 (PMC10927845; doi:10.3389/fresc.2024.1341740)
Supplement: Supplementary file 1 [file Table1.docx]

**Supplementary Material 1** Ovid MEDLINE(R) and In-Process, In-Data-Review & Other Non-Indexed Citations 1996 to February 27, 2023 Search Strategy

1. exp Resilience, Psychological/ or resilience.mp.

2. resilien*.mp.

3. 1 or 2

4. exp Developmental Disabilities/ or developmental disabilit*.mp. or exp Autism Spectrum Disorder/ or exp Intellectual Disability/

5. neurodevelopmental disabilit*.mp. or exp Intellectual Disability/ or exp Neurodevelopmental Disorders/

6. exp Tourette Syndrome/ or exp Attention Deficit Disorder with Hyperactivity/

7. exp Cerebral Palsy/

8. exp Brain Injuries/

9. exp Speech Disorders/

10. exp Language Development Disorders/

11. global developmental delay.mp.

12. Autism Spectrum Disorder/ or Autistic Disorder/

13. developmental coordination disorder.mp. or exp Motor Skills Disorders/

14. exp Disabled Children/ or childhood disability.mp. or exp Developmental Disabilities/

15. 4 or 5 or 6 or 7 or 8 or 9 or 10 or 11 or 12 or 13 or 14

16. exp Young Adult/ or exp Adolescent/ or adolescen*.mp.

17. exp Adolescent/

18. exp Adolescent/ or Teen*.mp.

19. puberty.mp. or exp Puberty/

20. 16 or 17 or 18 or 19

21. 3 and 15

22. 20 and 21

23. limit 22 to "all child (0 to 18 years)"
