# Supplementary material for: Resilience and adolescence-transition in youth with developmental disabilities and their families: a scoping review
Source: Front Rehabil Sci. 2024 Feb 27;5:1341740. doi: 10.3389/fresc.2024.1341740 (PMC10927845; doi:10.3389/fresc.2024.1341740)
Supplement: Supplementary file 2 [file Table2.docx]

**Supplementary Material 2** Resilience Factors Coding Scheme

The following was developed by the senior author based on recent and comprehensive knowledge synthesis and consensus projects related to child and adolescent mental health and resilience-focused interventions [1-4]. The coding scheme include four main resilience levels. These are individual (within the child / internal protective factors), family, school/peer, and community level factors (external protective factors).

| 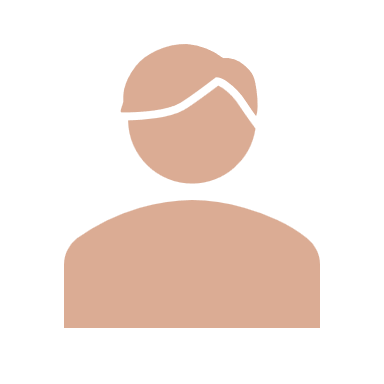**Individual**  **A** | 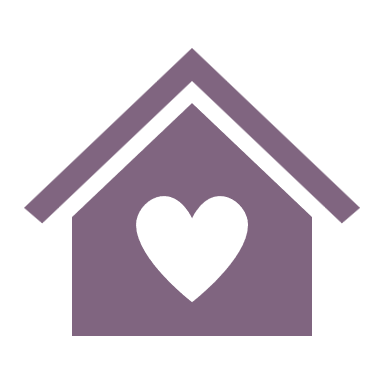**Family**  **B** | 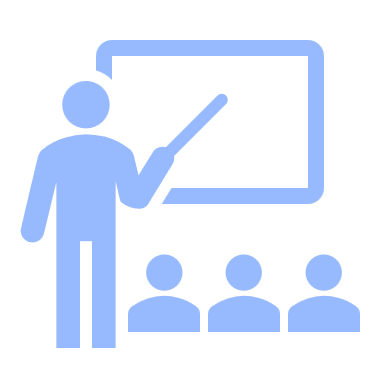**School and peers**  **C** | 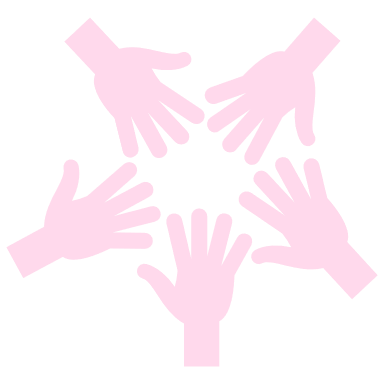**Community**  **D** |
| --- | --- | --- | --- |
| 1. Autonomy/independence 2. Cognitive competence 3. Cooperation and communication 4. Coping 5. Confidence 6. Culture 7. Emotional regulation 8. Empathy 9. Empowerment 10. Genetic and biological factors 11. Goals and aspirations 12. Healthy thinking habits 13. Hope 14. Moral competence 15. Motivation 16. Optimism and positive attitude 17. Problem solving and decision making 18. Spirituality 19. Self-control 20. Self-efficacy 21. Self-esteem 22. Self-regulation 23. Self-awareness 24. Self-talk/self-compassion 25. Self-reflection 26. Sense of responsibility/connection to family and community 27. Social and emotional competence 28. Social and emotional skills | 1. Home support 2. Home relationships (parents, siblings) 3. Home participation 4. Parenting style/skills 5. Culture/Spirituality | 1. Peer relationships and connection 2. Peer support 3. School support 4. School participation 5. School relationships 6. Culture | 1. Community relationships 2. Community support 3. Community participation 4. Public policies 5. Friendly spaces 6. Culture |

[1] J. Dray, "Child and adolescent mental health and resilience-focussed interventions: A conceptual analysis to inform future research," *International journal of environmental research and public health,* vol. 18, no. 14, p. 7315, 2021.

[2] J. Dray *et al.*, "Systematic review of universal resilience-focused interventions targeting child and adolescent mental health in the school setting," *Journal of the American Academy of Child & Adolescent Psychiatry,* vol. 56, no. 10, pp. 813-824, 2017.

[3] A. S. Masten, C. M. Lucke, K. M. Nelson, and I. C. Stallworthy, "Resilience in development and psychopathology: Multisystem perspectives," *Annual Review of Clinical Psychology,* vol. 17, pp. 521-549, 2021.

[4] B. Blue, "Building resilience in children aged 0-12-A practice guide," 2018.
