# Supplementary material for: Resilience and adolescence-transition in youth with developmental disabilities and their families: a scoping review
Source: Front Rehabil Sci. 2024 Feb 27;5:1341740. doi: 10.3389/fresc.2024.1341740 (PMC10927845; doi:10.3389/fresc.2024.1341740)
Supplement: Supplementary file 3 [file Table3.docx]

**Supplementary Material 3** Excluded citations of intervention and assessment studies by full text by reasons for exclusion

| No population of interest (n=52 studies; 11 intervention studies, 41 assessment studies) |
| --- |
| Andersen & Winther, 2023 [1] |
| Borek et al., 2018 [2] |
| Conaughton, Donovan & March, 2017 [3] |
| Cousineau, Hobbs & Arthur, 2019 [4] |
| Donnelly et al., 2021[5] |
| Iida et al., 2018 [6] |
| King et al., 2019 [7] |
| Wachpress, Maier & Mazor-Karsenty, 2019 [8] |
| Whiting et al., 2019 [9] |
| Wolford & Holtrop, 2020 [10] |
| Yaacob et al., 2022 [11] |
| Ambler, Medford & Hare, 2018 [12] |
| Bekhet, 2016 [13] |
| Brooks, 1994 [14] |
| Cho & Kim, 2022 [15] |
| Choi & Yoo, 2015 [16] |
| Choi & Van Riper, 2017 [17] |
| Cooper et al., 2021 [18] |
| Cosden, 2001 [19] |
| Darling Rasmussen et al., 2020 [20] |
| Dekker et al., 2022 [21] |
| Doege et al., 2011 [22] |
| Fuller et al., 2020 [23] |
| Giallo & Gavidia-Payne, 2006 [24] |
| Grant et al., 2013 [25] |
| Greeff, Vansteenwegen & Gillard, 2012 [26] |
| Hilliard et al., 2015 [27] |
| Jandasek et al., 2009 [28] |
| Lang et al., 2021 [29] |
| Levine, 2009 [30] |
| Manav, Marasli & Uymaz, 2021 [31] |
| Manicacci et al., 2019 [32] |
| Martel & Nigg, 2006 [33] |
| McCrimmon, Climie & Huynh, 2018 [34] |
| Missiuna et al., 2008 [35] |
| Modesto-Lowe et al., 2014 [36] |
| Montirosso et al., 2021 [37] |
| Poehlmann et al., 2005 [38] |
| Retzlaff, 2007 [39] |
| Robinson et al., 2018 [40] |
| Silverman et al., 2022 [41] |
| Simpson et al., 2022 [42] |
| Smith & Grzywacz, 2014 [43] |
| Szatmari, 2018 [44] |
| Timmons et al., 2016 [45] |
| Van Riper, 2007 [46] |
| Wang et al., 2022 [47] |
| Whitesell & Kaufman, 2017 [48] |
| Wilmshurst, Peele & Wilmshurst, 2011 [49] |
| Wintgens & Hayez, 2003 [50] |
| Yusuf et al., 2022 [51] |
| Zamani, Mychasiuk & Semple, 2019 [52] |
| No exposure of interest (n=20 studies; 3 intervention studies, 17 assessment studies) |
| Fell et al., 2022 [53] |
| Gan & Ballantyne, 2016 [54] |
| Kirchhofer et al., 2022 [55] |
| Bitsika, Sharpley & Mailli, 2015 [56] |
| Bunt et al., 2021 [57] |
| Deutz et al., 2018 [58] |
| Fourie & Theron, 2012 [59] |
| Gardiner, Masse & Iarocci, 2019 [60] |
| Gauvin-Lepage, 2022 [61] |
| Gauvin-Lepage, Lefebvre & Malo, 2015 [62] |
| Laliberte Durish, Yeates & Brooks, 2018 [63] |
| Lingam et al., 2011 [64] |
| Shapiro, 2002 [65] |
| Simoes, Matos & Morgan, 2015 [66] |
| Sutton et al., 2010 [67] |
| Suzuki et al., 2015a [68] |
| Szigeti et al., 2021 [69] |
| Ungar, 2004 [70] |
| Vaillant & Davis, 2000 [71] |
| Weber, 2016 [72] |
| Inability to obtain full text or publication type with missing information for extraction  (11 studies; n=4 intervention studies, 7 assessment studies) |
| Gauvin-Lepage, Lefebvre & Malo, 2013 [73] |
| Gauvin-Lepage, Lefebvre & Malo, 2016 [74] |
| Jacobs-Nzuzi Kuabi, Swart & Soeker, 2022 [75] |
| Lepage & Lefebvre, 2012 [76] |
| Choi, 2015 [77] |
| Frison, Wallander & Browne, 1998 [78] |
| Hechtman, 1991 [79] |
| Kersh, Siperstein & Moskowitz, 2012 [80] |
| Miranda et al., 2023 [81] |
| Retzlaff et al., 2006 [82] |
| Suzuki et al., 2015b [83] |

1. Andersen, M.M. and H. Winther, *'I Dare to Be Myself.' The Value of Peer Communities in Adapted Physical Activity Interventions for Young People and Adults with Cerebral Palsy.* Scandinavian Journal of Disability Research, 2023. **25(1)**: p. 1-14.

2. Borek, A.J., et al., *Healthy Parent Carers programme: development and feasibility of a novel group-based health-promotion intervention.* BMC Public Health, 2018. **18**(1): p. 270.

3. Conaughton, R.J., C.L. Donovan, and S. March, *Efficacy of an internet-based CBT program for children with comorbid High Functioning Autism Spectrum Disorder and anxiety: A randomised controlled trial.* J Affect Disord, 2017. **218**: p. 260-268.

4. Cousineau, T.M., L.M. Hobbs, and K.C. Arthur, *The Role of Compassion and Mindfulness in Building Parental Resilience When Caring for Children With Chronic Conditions: A Conceptual Model.* Front Psychol, 2019. **10**: p. 1602.

5. Donnelly, K.Z., et al., *A retrospective study on the acceptability, feasibility, and effectiveness of LoveYourBrain Yoga for people with traumatic brain injury and caregivers.* Disability & Rehabilitation, 2021. **43**(12): p. 1764-1775.

6. Iida, N., et al., *Effectiveness of parent training in improving stress-coping capability, anxiety, and depression in mothers raising children with autism spectrum disorder.* Neuropsychiatr Dis Treat, 2018. **14**: p. 3355-3362.

7. King, G., et al., *Solution-Focused Coaching in Pediatric Rehabilitation: Investigating Transformative Experiences and Outcomes for Families.* Physical & Occupational Therapy in Pediatrics, 2019. **39**(1): p. 16-32.

8. Wachspress, B., A. Maeir, and T. Mazor-Karsenty, *Content Validity of the Parentship Protocol: A Multidimensional Intervention for Parents of Adolescents with High-Functioning Autism Spectrum Disorder.* Physical & Occupational Therapy in Pediatrics, 2019. **39**(4): p. 373-387.

9. Whiting, M., et al., *Enhancing resilience and self-efficacy in the parents of children with disabilities and complex health needs.* Primary Health Care Research & Development, 2019. **20**: p. e33.

10. Wolford, S.N. and K. Holtrop, *Examining the Emotional Experience of Mothers Completing an Evidence-Based Parenting Intervention: A Grounded Theory Analysis.* Family Process, 2020. **59**(2): p. 445-459.

11. Yaacob, W.N.W., et al., *A Journey towards Resilience: Coping Strategies Adopted by Parents with Children Having Autism Spectrum Disorder in Northeast Malaysia.* International Journal of Environmental Research and Public Health, 2022. **19(4) (no pagination)**.

12. Ambler, O., E. Medford, and D.J. Hare, *Parenting a Child with Phenylketonuria: An Investigation into the Factors That Contribute to Parental Distress.* JIMD Rep, 2018. **41**: p. 91-100.

13. Bekhet, A.K., *The Mediating Effects of Positive Cognitions on Autism Caregivers' Depression and Their Children's Challenging Behaviors.* Archives of Psychiatric Nursing, 2016. **30**(1): p. 13-8.

14. Brooks, R.B., *Children at risk: fostering resilience and hope.* Am J Orthopsychiatry, 1994. **64**(4): p. 545-53.

15. Cho, Y. and H. Kim, *[A Predictive Model of Resilience in Mothers of Children with Developmental Disabilities].* Journal of Korean Academy of Nursing, 2022. **52**(4): p. 407-420.

16. Choi, E.K. and I.Y. Yoo, *Resilience in families of children with Down syndrome in Korea.* International Journal of Nursing Practice, 2015. **21**(5): p. 532-41.

17. Choi, H. and M. Van Riper, *Adaptation in families of children with Down syndrome in East Asian countries: an integrative review.* Journal of Advanced Nursing, 2017. **73**(8): p. 1792-1806.

18. Cooper, R., et al., *"I'm Proud to be a Little Bit Different": The Effects of Autistic Individuals' Perceptions of Autism and Autism Social Identity on Their Collective Self-esteem.* Journal of Autism and Developmental Disorders, 2021. **51(2)**: p. 704-714.

19. Cosden, M., *Risk and resilience for substance abuse among adolescents and adults with LD.* Journal of Learning Disabilities, 2001. **34**(4): p. 352-8.

20. Darling Rasmussen, P., et al., *Mapping factors facilitating resilience in mothers - potential clinical relevance for children with ADHD.* Nord J Psychiatry, 2020: p. 1-4.

21. Dekker, L., et al., *Impact of the COVID-19 pandemic on children and adolescents with autism spectrum disorder and their families: a mixed-methods study protocol.* BMJ Open, 2022. **12**(1): p. e049336.

22. Doege, D., et al., *[Resilience, coherence and stress in families of children with intellectual disabilities].* Praxis der Kinderpsychologie und Kinderpsychiatrie, 2011. **60**(7): p. 527-43.

23. Fuller, A.E., et al., *Relationships Between Material Hardship, Resilience, and Health Care Use.* Pediatrics, 2020. **145**(2): p. 02.

24. Giallo, R. and S. Gavidia-Payne, *Child, parent and family factors as predictors of adjustment for siblings of children with a disability.* Journal of Intellectual Disability Research, 2006. **50**(Pt 12): p. 937-48.

25. Grant, S., et al., *Parental social support, coping strategies, resilience factors, stress, anxiety and depression levels in parents of children with MPS III (Sanfilippo syndrome) or children with intellectual disabilities (ID).* Journal of Inherited Metabolic Disease, 2013. **36**(2): p. 281-91.

26. Greeff, A.P., A. Vansteenwegen, and J. Gillard, *Resilience in families living with a child with a physical disability.* Rehabilitation Nursing Journal, 2012. **37**(3): p. 97-104.

27. Hilliard, M.E., et al., *Resilience in youth and families living with pediatric health and developmental conditions: Introduction to the special issue on resilience.* Journal of Pediatric Psychology, 2015. **40**(9): p. 835-839.

28. Jandasek, B., et al., *Trajectories of family processes across the adolescent transition in youth with spina bifida.* J Fam Psychol, 2009. **23**(5): p. 726-38.

29. Lang, C.P., et al., *Children with cerebral palsy: A cross-sectional study of their sleep and their caregiver's sleep quality, psychological health and well-being.* Child: care, health and development, 2021. **47(6)**: p. 859-868.

30. Levine, K.A., *Against all odds: resilience in single mothers of children with disabilities.* Social Work in Health Care, 2009. **48**(4): p. 402-19.

31. Manav, G., B. Marasli, and P. Uymaz, *Assessment of depression levels, quality of life and care burden of parents with disabled children.* Current Pediatric Research, 2021. **25(12)**: p. 1191-1199.

32. Manicacci, M., et al., *Involvement of Emotional Intelligence in Resilience and Coping in Mothers of Autistic Children.* Journal of Autism & Developmental Disorders, 2019. **49**(11): p. 4646-4657.

33. Martel, M.M. and J.T. Nigg, *Child ADHD and personality/temperament traits of reactive and effortful control, resiliency, and emotionality.* Journal of Child Psychology & Psychiatry & Allied Disciplines, 2006. **47**(11): p. 1175-83.

34. McCrimmon, A.W., E.A. Climie, and S. Huynh, *The relation between emotional intelligence and resilience in at-risk populations.* Developmental neurorehabilitation, 2018. **21**(5): p. 326-335.

35. Missiuna, C., et al., *Life experiences of young adults who have coordination difficulties.* Canadian Journal of Occupational Therapy, 2008. **75(3)**: p. 157-166.

36. Modesto-Lowe, V., et al., *Parenting teens with attention-deficit/hyperactivity disorder: challenges and opportunities.* Clinical Pediatrics, 2014. **53**(10): p. 943-8.

37. Montirosso, R., et al., *Stress symptoms and resilience factors in children with neurodevelopmental disabilities and their parents during the COVID-19 pandemic.* Health Psychology, 2021. **40**(7): p. 428-438.

38. Poehlmann, J., et al., *Family experiences associated with a child's diagnosis of fragile X or Down syndrome: evidence for disruption and resilience.* Mental Retardation, 2005. **43**(4): p. 255-67.

39. Retzlaff, R., *Families of Children With Rett Syndrome: Stories of Coherence and Resilience.* Families, Systems and Health, 2007. **25(3)**: p. 246-262.

40. Robinson, S., et al., *Self-compassion and psychological distress in parents of young people and adults with intellectual and developmental disabilities.* Journal of Applied Research in Intellectual Disabilities, 2018. **31**(3): p. 454-458.

41. Silverman, M.R., et al., *Parental Factors That Confer Risk and Resilience for Remote Learning Outcomes During the COVID-19 Pandemic Among Children With and Without Attention-Deficit/Hyperactivity Disorder.* Journal of Attention Disorders, 2022. **26**(11): p. 1381-1393.

42. Simpson, T.S., et al., *Child, Parent, and Family Adjustment for Patients Followed in a Multidisciplinary Spina Bifida Clinic.* Top Spinal Cord Inj Rehabil, 2022. **28**(3): p. 41-58.

43. Smith, A.M. and J.G. Grzywacz, *Health and well-being in midlife parents of children with special health needs.* Families, Systems, & Health, 2014. **32**(3): p. 303-12.

44. Szatmari, P., *Risk and resilience in autism spectrum disorder: a missed translational opportunity?* Dev Med Child Neurol, 2018. **60**(3): p. 225-229.

45. Timmons, L., et al., *Predictors of Daily Relationship Quality in Mothers of Children with Autism Spectrum Disorder.* Journal of Autism & Developmental Disorders, 2016. **46**(8): p. 2573-2586.

46. Van Riper, M., *Families of children with Down syndrome: responding to "a change in plans" with resilience.* Journal of Pediatric Nursing, 2007. **22**(2): p. 116-28.

47. Wang, L., et al., *Mental health issues in parents of children with autism spectrum disorder: A multi-time-point study related to COVID-19 pandemic.* Autism Res, 2022. **15**(12): p. 2346-2358.

48. Whitesell, N.R. and C.E. Kaufman, *Substance Use Disorders Among Indigenous Youth in Developmental Perspective: Diversity, Diagnostic Tools, and Resilience.* Journal of the American Academy of Child & Adolescent Psychiatry, 2017. **56**(2): p. 103-104.

49. Wilmshurst, L., M. Peele, and L. Wilmshurst, *Resilience and well-being in college students with and without a diagnosis of ADHD.* Journal of Attention Disorders, 2011. **15**(1): p. 11-7.

50. Wintgens, A. and J.Y. Hayez, *The psychic life experience of siblings of children with mental disabilities or autistic disorders: Resiliency, adaptation or mental health troubles. [French].* Neuropsychiatrie de l'Enfance et de l'Adolescence, 2003. **51(7)**: p. 377-384.

51. Yusuf, A., et al., *Factors associated with resilience among children and youths with disability during the COVID-19 pandemic.* PLoS One, 2022. **17**(7): p. e0271229.

52. Zamani, A., R. Mychasiuk, and B.D. Semple, *Determinants of social behavior deficits and recovery after pediatric traumatic brain injury.* Experimental Neurology, 2019. **314**: p. 34-45.

53. Fell, L., et al., *Acceptability of A Virtual Mind-Body Group Intervention for Teen Siblings of Children with Autism Spectrum Disorder.* Journal of Autism & Developmental Disorders, 2022. **52**(12): p. 5243-5252.

54. Gan, C. and M. Ballantyne, *Brain injury family intervention for adolescents: A solution-focused approach.* Neurorehabilitation, 2016. **38**(3): p. 231-41.

55. Kirchhofer, S.M., et al., *A systematic review of social support for siblings of children with neurodevelopmental disorders.* Res Dev Disabil, 2022. **126**: p. 104234.

56. Bitsika, V., C.F. Sharpley, and R. Mailli, *The influence of gender, age, Psychological resilience and family interaction factors upon anxiety and depression in non-autism spectrum disorder siblings of children with an autism spectrum disorder.* British Journal of Guidance & Counselling, 2015. **43**(2): p. 216-228.

57. Bunt, S.C., et al., *Resilience and recovery from sports related concussion in adolescents and young adults.* Journal of Clinical & Experimental Neuropsychology: Official Journal of the International Neuropsychological Society, 2021. **43**(7): p. 677-688.

58. Deutz, M.H.F., et al., *Evaluation of the Strengths and Difficulties Questionnaire-Dysregulation Profile (SDQ-DP).* Psychological Assessment, 2018. **30**(9): p. 1174-1185.

59. Fourie, C.L. and L.C. Theron, *Resilience in the face of fragile X syndrome.* Qualitative Health Research, 2012. **22**(10): p. 1355-68.

60. Gardiner, E., L.C. Masse, and G. Iarocci, *A psychometric study of the Family Resilience Assessment Scale among families of children with autism spectrum disorder.* Health & Quality of Life Outcomes, 2019. **17**(1): p. 45.

61. Gauvin-Lepage, J., *Traumatic Brain Injury in Adolescence and the Family Resilience Process: A Case Study.* Brain Injury, 2022. **36(SUPPL 1)**: p. 11-12.

62. Gauvin-Lepage, J., H. Lefebvre, and D. Malo, *Resilience in Families With Adolescents Suffering From Traumatic Brain Injuries.* Rehabilitation Nursing Journal, 2015. **40**(6): p. 368-77.

63. Laliberte Durish, C., K.O. Yeates, and B.L. Brooks, *Convergent and divergent validity of the Connor-Davidson Resilience Scale in children with concussion and orthopaedic injury.* Brain Injury, 2018. **32**(12): p. 1525-1533.

64. Lingam, R.P., et al., *Identity and empowerment: A qualitative study of teenagers with developmental coordination disorder.* Archives of Disease in Childhood, 2011. **1)**: p. A7-A9.

65. Shapiro, E.R., *Chronic illness as a family process: a social-developmental approach to promoting resilience.* Journal of Clinical Psychology, 2002. **58**(11): p. 1375-84.

66. Simoes, C., M.G. Matos, and A. Morgan, *Facing the Adversity: the Role of Internal Assets on Well-Being in Adolescents with Special Needs.* Spanish Journal of Psychology, 2015. **18**: p. E56.

67. Sutton, M.B., et al., *Resilience, childhood disability and the Internet--case report.* Wiener Medizinische Wochenschrift, 2010. **160**(13-14): p. 325-7.

68. Suzuki, K., et al., *Development and Evaluation of a Parenting Resilience Elements Questionnaire (PREQ) Measuring Resiliency in Rearing Children with Developmental Disorders.* PLoS One, 2015. **10**(12): p. e0143946.

69. Szigeti, Z., et al., *The Road to Family Resiliency: A Case Report of a Family's Experiences Following Adolescent Stroke.* Rehabilitation Nursing Journal, 2021. **46**(2): p. 87-94.

70. Ungar, M., *The importance of parents and other caregivers to the resilience of high-risk adolescents.* Family Process, 2004. **43**(1): p. 23-41.

71. Vaillant, G.E. and J.T. Davis, *Social/emotional intelligence and midlife resilience in schoolboys with low tested intelligence.* American Journal of Orthopsychiatry, 2000. **70**(2): p. 215-22.

72. Weber, C.L., *Understanding fragile X syndrome from a mother's perspective: Challenges and resilience.* International Journal of Qualitative Studies on Health and Well-being, 2016. **11**: p. 29512.

73. Gauvin-Lepage, J., H. Lefebvre, and D. Malo, *Resilience in families of adolescents with traumatic brain injuries: Development of a support intervention.* Archives of Physical Medicine and Rehabilitation, 2013. **94(10)**: p. e40.

74. Gauvin-Lepage, J., H. Lefebvre, and D. Malo, *Resilience in families of adolescents with traumatic brain injuries: Development of a support intervention.* Brain Injury, 2016. **30(5-6)**: p. 552-553.

75. Jacobs-Nzuzi Khuabi, L.A.J., E. Swart, and M.S. Soeker, *Towards occupational resilience: A model to facilitate high school participation post traumatic brain injury.* Work (Reading, Mass.), 2022. **72(2)**: p. 463-482.

76. Lepage, J.G. and H. Lefebvre, *Resilience in families of adolescents with severe traumatic brain injuries: Development of a support intervention.* Brain Injury, 2012. **26(4-5)**: p. 315-316.

77. Choi, H., *[Adaptation in Families of Children with Down Syndrome: A Mixed-methods Design].* Journal of Korean Academy of Nursing, 2015. **45**(4): p. 501-12.

78. Frison, S.L., J.L. Wallander, and D. Browne, *Cultural factors enhancing resilience and protecting against maladjustment in African American adolescents with mild mental retardation.* American Journal of Mental Retardation, 1998. **102**(6): p. 613-26.

79. Hechtman, L., *Resilience and vulnerability in long term outcome of attention deficit hyperactive disorder.* Can J Psychiatry, 1991. **36**(6): p. 415-21.

80. Kersh, J., G.N. Siperstein, and A.L. Moskowitz, *The well-being of parents of adolescents with IDD: Results from the Special Olympics Family Resiliency Study.* Journal of Intellectual Disability Research, 2012. **56(7-8)**: p. 733.

81. Miranda, A., et al., *[Risk/resilience factors in families with children with autism. Association with evolution in adolescence].* Medicina (B Aires), 2023. **83 Suppl 2**: p. 53-57.

82. Retzlaff, R., et al., *[Family sense of coherence and resilience. A study on families with children with mental and physical disabilities].* Praxis der Kinderpsychologie und Kinderpsychiatrie, 2006. **55**(1): p. 36-52.

83. Suzuki, K., et al., *[Parenting resilience for rearing a child with autism spectrum disorder: a qualitative study].* No to Hattatsu [Brain & Development], 2015. **47**(4): p. 283-8.
