# Supplementary material for: Resilience and adolescence-transition in youth with developmental disabilities and their families: a scoping review
Source: Front Rehabil Sci. 2024 Feb 27;5:1341740. doi: 10.3389/fresc.2024.1341740 (PMC10927845; doi:10.3389/fresc.2024.1341740)
Supplement: Supplementary file 4 [file Table4.docx]

**Supplementary Material 4A:** Resilience definitions and Models/Framework Used – Observation studies

| **Reference** | **Resilience Definition** | **Model/Framework** |
| --- | --- | --- |
| **ASD** | | |
| Bekhet et al. [1] | “According to resilience theory (Van Breda, 2001), resilience is a dynamic process of balancing risk and protective factors in the face of adversity (Luthar, Cicchetti,& Becker, 2000)”. | No framework or model is specified |
| Bekhet [2] | No definition for resilience provided | The study is informed by Goodman and Gotlib (1999), integrative model for understanding children's risk in relation to maternal depression. According to Goodman and Gotlib's theory, one of the identified risks of child's psychopathology is the child's exposure to negative maternal cognitions, behaviors, and affect.  The study is also informed by Karazsia and Wildman (2009) conceptual model of maternal affect, and children's behavior (Karazsia & Wildman, 2009). According to Karazsia and Wildman's model, maternal affect has a direct influence on the child's behavior problems. |
| Bayat [3] | Resilience has been described as the ability to withstand hardship and rebound from adversity, becoming more strengthened and resourceful (Walsh 1998). | The theoretical framework used for categorization of thematic data in this study is the **Walsh’s** (1998, 2003) theory, which considers the following components as those belonging to resilient families: (1) making meaning of adversity, (2) affirming strength and keeping a positive outlook, and (3) having spirituality and belief system. |
| Bitsika [4] | Psychological resilience refers to an individual’s capacity to resist the harmful effects of adverse stressors and to resume functioning despite them (Luthar & Cicchetti, 2000). | No framework or model is specified |
| Bitsika [5] | Psychological resilience refers to an individual’s capacity to cope with stressors and to resist the harmful effects of those stressors (Luthar & Cicchetti, 2000). Various definitions of resilience have been offered, including a personal trait that promotes rebounding from disappointments (Brooks, 2005), positive adjustment in adverse circumstances (Tedeschi & Kilmer, 2005) or successful adaptation to challenging life stressors (Alvord & Grados, 2005). | No framework or model is specified |
| Fong et al. [6] | Family resilience is defined as the ‘characteristics, dimensions, and properties which help families to be resistant to disruption in the face of change and adaptive in the face of crisis situations’ (McCubbin & McCubbin, 1988, p. 247). | Data used for the current analysis originated from a larger mixed methods study.  Furthermore, theoretical foundations advanced by Bronfenbrenner’s (1979) **ecological model**  **and Family Systems Theory** (Turnbull et al., 1984) have guided empirical studies on resilience and have provided a framework for the development of interventions for children with disabilities and their families (Gardiner & Iarocci, 2012). |
| Hayes et al. [7] | Family resilience and connection were significantly associated with child flourishing, and the strength of the relationship was not significantly impacted by exposure to adversity. | Resilient families often exhibit belief systems that foster healing as well as the ability to make meaning of and openly communicate about adverse experiences (Walsh, 2003). Specifically, family resilience includes having a positive outlook, sharing decision- making and talking through problems as a family. Family resilience has been consistently associated with positive child outcomes, caregiver well-being and family quality of life (Bethell et al., 2019; Leone et al., 2016). |
| Iannuzzi et al. [8] | No definition for resilience provided | Mention of a recent systematic review of qualitative literature by Leedham and colleagues included 18 studies focused on the lived experience of NT siblings over the lifespan. Common themes reported include the impact of aggressive and unpredictable behaviors, embarrassment and the feeling of “being different” than peers, and lessons learned about acceptance and empathy. |
| King et al. [9] | Resilience is a dynamic process of successful adaptation despite challenging or adverse circumstances, which acknowledges both hardship and benefit (Luthar et al. 2000). Parents’ perceptions of positive contributions can be seen as part of this adaptation (Hastings et al. 2002). | Use of a **grounded theory approach**, where the aim is to organize concepts and relationships in the data into a theoretical explanatory scheme (Strauss & Corbin 1998). |
| McCarthy et al. [10] | Resiliency reflects the degree to which one's personal re- sources (mastery and relatedness) match or exceed their emotional reactivity to stress (Masten, 2004; Prince-Embury, 2006, 2007). | Basis on two distinct theoretical models of emotional intelligence (EI) which have been proposed; the **ability model** defines EI as a set of emotion-related cognitive abilities (Mayer, Caruso & Salovey, 1999) assessed through performance measures (Mayer, Caruso & Salovey, 2016). Conversely, the **trait model** defines EI as a constellation of emotional perceptions assessed through questionnaires (Petrides, Pita & Kokkinaki, 2007). Greater trait EI has been associated with greater mastery and relatedness, and lower emotional reactivity in typically developing children (8 to 12 years; McCrimmon, Climie & Huynh, 2018, 2016), adolescents (14 to 16 years; ; Droppert et al., 2019), and young adults (18 to 25 years; Di Fabio & Saklofske, 2018), suggesting that individuals with well-developed EI are more resilient. |
| McConnell et al. [11] | Resilience is defined here as positive family adaptation in response to, or despite exposure to child behavior problems | This study was nested within a three year—survey plus interview—study of work-family-care integration in families bringing up children with disabilities in Alberta, Canada. The analysis reported in this paper is based on Year 1 survey data.  Within-family and social–ecological factors are inter-linked in **ecocultural theory.** A central tenet of this theory is that families at once shape and are shaped by the social–ecological context in which they live (Gallimore, Weisner, Bernheimer, Guthrie, & Nihira, 1993; Gallimore, Bernheimer, & Weisner, 1999; Weisner, Matheson, Coots, & Bernheimer, 2005). This theory posits that all families face the same enduring adaptive challenge: to create and maintain a sufficiently predictable daily routine, or way of life, that is congruent with their values and aspirations, and the needs, interests and competences of individual family members. |
| McCrimmon et al. [12] | Resilience describes the capacity of individuals to demonstrate positive outcome despite experiencing adversity or  trauma [46]. At its most fundamental, the construct of  resilience strives to explain the inherent and environmental  protective and risk factors related to developmental outcome. | Mention of the ‘Theory of Mind’’ (ToM), the ability to conceive of another’s mental states such as their knowledge, wants, feelings, and beliefs, has been proposed to underlie the sociocommunicative impairments observed in individuals with  HFASD |
| Menezes et al. [13] | Resilience can be defined as “a dynamic process encompassing positive adaptation within the context of significant adversity” (Luthar et al., 2000, p. 545), and it is applicable to both individuals and systems, such as families (Masten, 2014; Patterson, 2002). While family resilience is not a static trait and its demonstration may differ based on specific family and contextual factors, it can broadly be understood as the process of leveraging collective strengths in times of particular stress and ultimately, achieving some metric of success. | Data from this study were obtained from three waves of the National Survey of Children’s Health (NSCH) – 2016, 2017, and 2018.  Mention of results from a study conducted by Halstead et al. (2018) suggest that resilience may be an independent predictor of caregiver coping in families with children and adolescents with ASD. Halstead et al. (2018) found that maternal resilience functioned as a compensatory factor of well-being in mothers of children with developmental disability and autism; maternal resilience had a significant main effect relationship with maternal well-being outcomes |
| Montes & Halterman [14] | No definition for resilience provided | Based on the National Survey of Children’s Health (NSCH) which is part of the State and Local Area Integrated Telephone Survey program conducted by the National Center for Health Statistics. The survey interviewed 102 353 parents of children who were aged 0 to 17 between January 2003 and July 2004 (87% of the interviews were completed in 2003). |
| Nahar et al. [15] | Resilience is defined as a “process wherein individuals display positive adaptation despite experiences of significant adversity or trauma” (Luthar & Cicchetti, 2000, p. 858). Resilience may act as a protective factor against negative outcomes associated with raising a child with ASD, such as increased anxiety and depression (Bitsika et al., 2013), with positive associations with parental mental health, including lower levels of depression (Ekas et al., 2010). | Mention of a study conducted by Blacher and McIntyre (2006) and Hastings (2008) who concluded that when behavioural problems of children with ASD are controlled for, the psychological well-being of parents is no different from parents of other populations. This may indicate the presence of ASD itself may not be a factor for these differences, but rather the associated behavioural difficulties in children.  Additional mention of two coping styles; problem-focused coping (e.g. planning, taking-action), and emotion-focused coping (e.g. denial, wishful thinking) (Folkman & Lazarus, 1986). Coping styles have been reported to be a moderator between stressors (e.g. life events) and negative outcomes (e.g. depression) among parents of children with ASD (Dunn et al., 2001). Therefore, the literature has indicated the type of coping style utilised is associated with the variation in parental psychological outcomes. |
| O’Brien [16] | Resiliency, meaning the ability of families to rebound from difficulties or upheavals. | **The Resiliency Model of Family Adjustment and Adaptation (RMF)** (McCubbin et al., 2003) served as the study's framework. This model is frequently utilized by nurses and social scientists to promote resiliency. The model's outcome is family adaptation or balance, which may be disrupted after one catastrophic crisis or after a pile-up of demands, stressors, strains, and life transitions  The RMF model is an appropriate model for studies regarding families of children with disabilities. Although all families experience cycles of adjustments and upheavals, including normal developmental changes, families of children with chronic conditions are impacted more deeply by these challenges (Patterson, 1988). |
| Picardi et al. [17] | No concrete definition for resilience provided | Framework of the ‘Ricerca Finalizzata 2007’ program.  Mention of ‘resilient disruption’ model of family adaptation developed to account for the impressive resilience of many families to the stress associated with disability, which posits that families are both disrupted by and resilient to the stress associated with raising a child with disability. |
| Ruiz-Robledillo et al.  [18] | This definition refers to resilience as a way of coping with stress in an adaptive manner. More specifically, resilience in a care context has been defined as the ability shown by caregivers to bounce back from the stress derived from a care situation. In this sense, resilience in caregivers is configured by specific skills that promote a successful adaptation to a care situation without health being affected | Brief mention of one study carried out with caregivers of people with ASD which found a positive relationship between social support and CAR (cortisol awakening response) (Lovell et al., 2012b) |
| Schneider et al. [19] | The concept of resilience, which can be defined as the ability to “bounce back” or positively adapt to adversity. In addition to internal sources (e.g., flexibility, temperament, intelligence, sociability) that can affect resilience, resilience during childhood is highly dependent on external sources, such as family/support systems that care for the child and access to appropriate resources within the larger system. The concept of family resilience, the family’s ability to cope and adapt to change, stress, or adversity, may be especially crucial in bouncing back from adversities during childhood. | Based on **Walsh theory of family resilience**, 4 items of family resilience were used in this study. Specifically, parents endorsed the family’s ability to talk about problems, know they have strengths to draw on, work together to solve problems, and remain hopeful in difficult times. Parents marked items on a 1 (All the time) to 4 (None of the time) scale, which was reverse coded. Items were summed to create a composite score of family resilience score ranging from 4 to 16, where higher scores indicated more family resilience. |
| Szatmari et al. [51] [20] | Over time the notion of resilience has been refined and now has more contextual nuance; resilience is seen not only as a characteristic of the child but also as a potential characteristic of the environment that strengthens the child’s ability to cope with adversity (Ungar, Ghazinour, & Richter, 2013).  A more nuanced appreciation of resilient “outcomes” is also emerging. A child might be resilient regarding a particular outcome (eg, not developing a mental disorder) but not another (eg, poor school performance or poor ability to form social attachments) (Masten, 2014). In addition, resilience must be seen in a developmental context. A child can be resilient at one time point (eg, in early childhood) and not at another (eg, later in adolescence) (Masten, 2014). | Mention of the Wehmeyer theory of self-determination. The concept of self-determination refers to the development of skills, attitudes, and beliefs that enable individuals to exercise control over their own lives (Wehmeyer, Abery, Mithaug, & Stancliffe, 2003).  Mention of Havighurst’s framework pertaining to developmental health. Havighurst (1972) proposed that the stages of human development may be best conceptualized as a series of integrated tasks. Within this framework, a “resilient” individual is one who has good developmental health and who, having experienced some adversity, is still able to meet the demands of a specific developmental stage. |
| Tomeny [21] | No concrete definition for resilience provided | Mention of previous research by Davis and Carter, 2008; Ingersoll and Hambrick, 2011; Sawyer et al., 2010; Taylor and Warren, 2012 |
| Zhao & Fu [22] | Parental resilience—the dynamic process of being able to parent effectively in the face of having a child with ASD  The study of resilience is evolving from individualistic conceptualizations to family resilience focusing on relational resilience in the family (McCubbin and McCubbin 2005, Walsh 2003). | Perspective of **social ecological theory** (Sun and Stewart 2007). Framework of the development of resilience in parents of children ASD.  Three themes by finding the interactions among the categories according to the social ecosystems theory: the interaction within the micro system, the interactions between the micro system and mezzo system, the interaction between micro systems and macro systems. |
| **ADHD** | | |
| Chan et al. [23] | Resilience from a positive youth development perspective is conceptualized as a dynamic interaction between the individual and context (Lerner et al., 2013; Masten, 2014a). Importantly, resilience requires both an experience of risk or vulnerability to negative outcomes (e.g., presence of ADHD), and a pattern of positive adaptation in the context of that adversity (Lerner, 2009; Masten & Obradovic, 2006). Resilient children are broadly characterized as exhibiting a diverse set of behaviors, skills, and attributes (Alvord & Grados, 2005) that include engaging in prosocial behaviors or seeking resources (Sanders et al., 2015), using problem solving skills (Coşkun, Garipağaoğlu, & Tosun, 2014), and harnessing family and/or social community assets to cope with challenges and recover from setbacks (Masten, 2014a). | Reflection of Lee, Sibley, and Epstein (2015); described as an initial step in this line of research derived from these authors’ framework |
| Dvorsky et al. [24] | No definition for resilience provided | Developed in relation to Ren et al.’s 2021 study exploring depressive symptoms during school closures in spring 2020 and Slik et al.’s 2021 study focusing on the effect of the pandemic and online schooling on adolescent girls experiencing depressive/anxiety symptoms and negative affect  Participants from the **larger study (Becker et al., 2019; Langberg et al., 2019)** who provided permission for further contact pre-COVID-19 (visits between September 2018 and February 2020; N = 262; 90.8% retention) were invited to participate in the current study, with COVID-19 data being collected between May 15 and June 14, 2020 during initial stay-at-home orders (spring 2020), between July 1 and August 5, 2020 (summer 2020), and between October 15 and November 19, 2020 (fall 2020). |
| Dvorsky et al.  [25] | Resilience models suggest that there are likely to be multiple trajectories of self-worth and that despite experiencing impairment, some youth with attention deficit/hyperactivity disorder (ADHD) may maintain a positive self-worth, which could buffer them against negative outcomes. | **Theoretical models:** Cognitive-behavioral (Safren, Sprich, Chulvick, & Otto, 2004), competency-based (Cole, 1990), and interpersonal (Ybrandt, 2008) models each theorize that self-worth plays an important role in the development of socioemotional and academic functioning.  The cognitive-behavioral model of ADHD emphasizes the role of repeated failure experiences with specific tasks (e.g., homework completion) in youth developing negative thought patterns and self-schemas, such as viewing themselves as incompetent (Barber, Grubbs, & Cottrell, 2005; Knouse & Safren, 2010; Wehmeier et al., 2010). |
| Dvorsky et al. [26] | Resilience is a broad term that reflects “positive patterns of adaptation in the context of adversity” (Masten & Obradovic, 2006, p. 14). | **Developmental models of risk-resilience** have emphasized the longitudinal progression of early problem behavior as antecedents for future impairment including academic failure and school dropout (e.g., Kazdin, Kraemer, Kessler, Kupfer, & Offord, 1997; Tremblay et al., 1992). It is important that behavior problems (e.g., inhibition, impulsivity, aggression) identified as early as preschool and elementary school predict academic problems in adolescence (e.g., Gray, Carter, Briggs-Gowan, Jones, & Wagmiller, 2014). For example, Kupersmidt and Coie (1990) found that preadolescent externalizing behavior predicted high school dropout even after controlling for peer rejection and absenteeism. |
| Mikami et al. [27] | No concrete definition for resilience provided | For details of the summer program methodology and initial cross-sectional findings, see  Hinshaw (2002).  Mention of **risk-resilience model** that may predict positive adjustment  for girls with the risk factors of ADHD and peer rejection (Masten, 2001)  Supporting this theory, research has found that girls who have little social support to counteract media pressures for thinness are vulnerable to eating pathology (Stice, Spangler, & Agras, 2001). Additionally, girls with ADHD may be at particular risk for eating pathology because of the impulsivity that is central to both ADHD and bulimia/binge eating disorders (Fahy & Eisler, 1993). |
| Ray et al. [28] | No concrete definition for resilience provided | **Risk-resilience model** for social functioning, including testing compensatory (i.e., main; buffering) and protective (i.e., inter- action) effects of enhancers in the presence of identified risk factors. Youth conduct problems, youth depression, and negative parenting emerged as risk factors. |
| Regalla et al. [29] | Resilience, the ability to overcome and recover from challenges, has been scarcely investigated in ADHD and could potentially provide novel strategies for treatment.  Resilience is defined as the ability to overcome adversity and become stronger – a process of resistance and growth following a crisis or challenge. | Mention of a study by Wilmshurst et al. which examined psychological well being, self-concept, and academic performance in ADHD-diagnosed college students and found significant differences compared to a control group. |
| Regalla et al. [30] | Resilience can be defined as a dynamic process through which a person thrives/copes/deals with events and risks in the context of his personal characteristics, familial, social and cultural history. | Brief mention of a study conducted by Dvorsky & Langberg wherein social and family factors comprised the most contributing factors promoting resilience in ADHD |
| Song et al. [31] | The variation in outcomes among youth exposed to risk factors can be explained by resilience, defined as “positive adaptation in the context of risk or adversity…(encompassing) a range of phenomena, including the capacity for doing well under adversity, the processes of coping with challenges, recovery from catastrophe, posttraumatic growth, and the achievement of good outcomes among people at high risk for failure or maladaptation” (Masten, 2014, p. 9) | Mention of a study using the National Child Traumatic Stress Network’s (NCTSN) Core Data Set (CDS) found that each additional type of traumatic exposure was associated with increased odds of aggressive behavior and attention problems for youth with ADHD (Greeson et al., 2014).  Mention of a systematic review of resilience-based studies of youth with ADHD identified 9 studies of familial characteristics’ protective and/or promotive effects on youth outcomes (Dvorsky and Langberg, 2016). Two of the 9 studies examined outcomes pertaining to behavioral and psychological functioning for ADHD-only adolescent samples; one study was longitudinal with person-and variable-centered methodology (Chronis et al., 2007) and the other cross-sectional and variable-centered (Schei et al., 2015). The two studies supported both promotive (i.e., direct) and protective effects (i.e., buffering the risks posed by ADHD symptom severity) of positive parenting on conduct problems and family cohesion on quality of life, respectively. As noted previously, family cohesion is associated with youths’ ability to communicate about and cope with problems (Meadows et al., 2006); this may be the mechanism by which family cohesion buffers the challenges posed by symptoms (e.g., inattentiveness, impulsivity) ADHD adolescents encounter. |
| Uddin et al. [32] | No concrete definition for resilience provided | Focus on **stress buffering models** suggest that secure family functioning and  parental attachment can buffer the deleterious effects of psychosocial  and traumatic stress exposure on physical and mental health  (Balistreri and Alvira-Hammond, 2016; Banyard et al., 2001;  Masten et al., 1999)  Mention of **psychopathological models** theorizing that  family violence and dysfunctional relationships could produce parental  affective disorders, depression, and anxiety, and in turn, contribute to  the development of internalizing and externalizing behavioral outcomes among children (Bagner et al., 2009; Schachar, 1991). |
| Ünver & Arman & Akpunar [33] | The term “emotional resilience” is expressed as the capacity to use coping skills (such as changing the schema of thinking, diverting attention to something else, seeking support, and looking for new ways) in regulating the intense negative emotions that children feel in the face of adverse life events | Focus on the development of metacognitive processes between the ages of 3 and 5 years in addition to the **development of theory of mind** and can continue for a lifetime.  Theory of mind includes metacognitive skills as a social-cognitive skill that  includes the ability to think about the mental states  of oneself and also others |
| **TBI** | | |
| Durish et al. [34] | Psychological resilience is conceptualized as interpersonal qualities that enable one to successfully adapt to adverse events, such as illness or injury. It is comprised of factors such as tolerance of negative affect, positive acceptance of change, perceived control, and personal competence (Conner & Davidson, 2003). | No framework or model is specified |
| Ernst et al. [35] | Psychological resilience can be defined as a mental and behavioral process which utilizes available assets to protect the self from stressors (Fletcher & Sarkar, 2013). Stated very simply, resiliency is the ability to “bounce back” following a potentially stressful or negative event (Smith et al., 2008). | Mention of one recent study which retrospectively identified pediatric participants (aged 8–18 years) who sustained either concussions or orthopedic injuries at least 6 months prior. Low |
| Laliberté Durish et al. [36] | Psychological resilience is conceptualized as the process of harboring interpersonal qualities that enable one to adapt or thrive in the face of adversity. Such qualities include personal competence, tenacity, trust in one’s instincts, and positive acceptance of change (Connor & Davidson, 2003). Psychological resilience can also be thought of as one’s ability to “bounce back” from illness, injury, or other stressors. | Mention of a larger parent project which equally took place in Calgary, AB. |
| Tonks et al. [37] | Resilience refers to attributes associated with hardiness, self-esteem, social skill, optimism, competence or achievement [18]. It is commonly associated with ‘bouncing back’ from adversity or trauma. | Protective model: In contrast to ‘deficit’ models of post-brain injury outcome, a greater focus on protective factors that promote and support resilience may be helpful for those assessing and treating children with ABI.  Mention of a pilot study testing materials  Inclusion of conceptual model: Mediation model—resilience is mediated by executive functioning ability. |
| **DD** | | |
| Greeff & Nolting [38] | Defines family resilience as the “characteristics, dimensions, and properties which help families to be resistant to disruption in the face of change and adaptive in the face of crisis situations.” Family resilience is not a static set of strengths that renders a family resilient, but rather an adaptive pathway, unique to each family and situation (McCubbin & McCubbin, 1988, p. 247). | **Walsh** (2003) identified **three domains of family functioning**— namely, family belief systems, organizational patterns, and communication processes—which can facilitate the reduction of stress, foster growth, and empower families to overcome adversity, that is, to become resilient.  **McCubbin & McCubbin (1996)** proposed the resiliency **model of family stress, adjustment, and adaptation**, which provides a theoretical framework outlining the processes involved in a family’s response to a stressor |
| Harrowell et al. [39] | No definition for resilience provided | Follow-up study involving the Avon Longitudinal Study of Parents and Children (ALSPAC) cohort  The current study tests the Environmental Stress Hypothesis, which considers poor motor coordination to be the primary stressor, which leads to multiple secondary stressors, such as frustration at school, negative peer inter- actions, or bullying, which, in turn, contribute to poor self-esteem and subsequently internalizing problems. Conversely, protective factors, such as social support, guard against this cascade.  *A ‘variable-focused model’ of resilience was used to consider how different variables influenced the risk of psychopathology in children with DCD compared with controls. |
| Janssen et al. [40] | No definition for resilience provided | Mention of earlier studies in this field, summarised by Dickinson et al. which relied heavily on parent reports or proxy reports, or focused on functions, symptoms and activities as indirect indicators. Additional mention of an exploratory study with a small sample of parents of children with CP found that symptoms of internalizing problems, such as anxiety, withdrawal and depressed affect, were negatively related to measures of QoL in children with CP. |
| Lingam et al. [41] | No definition for resilience provided | Mention of Fitzpatrick and Watkinson (2003) highlighted the difficulties experienced by a self-selected group of adults (aged 30–60 years) who described themselves as physically awkward (Fitzpatrick & Watkinson 2003). Emphasis was placed on the humiliation caused by frequent failures in physical tasks (Fitzpatrick & Watkinson 2003). These findings are in contrast to those by Missiuna et al. (2008) interviewing a group of young adults, recruited from university and college, who emphasized the development of strategies they had developed to overcome difficulties (Missiuna et al. 2008).  Mention of Bury’s ‘socio-medical model of disabling illness’, which considers both the impact of the illness or impairment on the individual but also the role of society in disability (Bury 1997). This is similar to previous work looking at British, Canadian and Swedish children with different impairments (Shakespeare et al. 1999; Mundhenke et al. 2009; Shikako- Thomas et al. 2009). |
| Schuengel et al. [42] | No definition for resilience provided | Harter scales for self-worth and perceived competence: According to Harter [3], two types of cognitions about the self are important: global sense of self-worth (often also referred to as self-esteem) and perceived competence within distinct domains of functioning. |
| Stang et al. [43] | No concrete definition for resilience provided | Using **grounded theory,** a codebook related to bullying was developed by three female authors independently (Strauss & Corbin, 1994). One author is an MD candidate, one is a DO, and the third is a PhD, RN. The coders were not actively involved in the original pilot study. Common responses were assigned keywords. The authors met to consolidate keywords into categories of shared experiences. After an iterative process, key codes, definitions, and examples were identified (Starks & Trinidad, 2007). The research team was debriefed |
| Widyawati et al. [44] | By definition, resilience is the process of the parents’ adaptation and adjustment to difficult family situations and the manner in which they become strengthened, resourceful and confident in handling difficult situations (Suzuki et al., 2013). | From the theoretical point of view, the indirect effects of positive perception of parenting can  be explained as follows. A positive perception of parenting enables parents to reframe and accept their children’s condition, which results in positive parenting. |
| Woodman & Hauser-Cram [45] | No concrete definition for resilience provided | Mention of the **buffering model of coping** states that coping strategies will have a greater impact on parent well-being under conditions of high stress (Essex et al. 1999). In contrast to the buffering hypothesis, the **direct effects model** predicts that coping will have the same effect on parental well-being regardless of level of behaviour problems (Ensel & Lin 1991). |
| **ID** | | |
| Hall & Theron [46] | Resilience, or the process of adjusting well to risk, relies on constructive collaboration between youths and their social ecologies. | **The Social Ecology of Resilience Theory** (SERT; see Ungar, 2011), which forms the theoretical framework of this article, accentuates the interactive nature of resilience. SERT explains that functional out- comes result when individuals and their social systems collaborate in ways that support positive adjustment to adversity. This includes individuals making use of, and/or negotiating for, assistance that supports functional outcomes, and social ecologies providing, and advocating for, accessible resilience-supporting resources (Ungar, 2015). Such collaboration is optimal when it occurs in contextually relevant ways, given that resilience is a contextually sensitive process (Ungar, Ghazinour, & Richter, 2013). |
| Hauser et al. [47] | No definition for resilience provided | **Family systems theory** suggests that the trends identified regarding maternal mental health, child behavior, and family environment for families with FXS are significant because all three aspects of family functioning have the potential to influence one another (von Bertalanffy, 1968). Because familial relationships exist as a system, the experience of one family member affects all members of the family in some way. This framework provides a rationale for examining the bidirectional relationship between maternal mental health status and child behavioral outcomes, as well as for examining the effects of both of these factors on the overall family environment. Such data are critical for identifying the factors that affect the family system most powerfully and that should be targeted by clinical intervention. |
| Jordan et al. [48] | No definition for resilience provided | Data were collected as part of a longitudinal study supported by the National Institute of Health examining cognition, behavior, and neurodevelopment in school-aged girls with FXS and their sex-, age-, and developmentally matched peers (see Bartholomay et al., 2019; Miller et al., 2021 for preliminary findings from this larger study). |
| Lloyd & Hastings [49] | No concrete definition for resilience provided | While this study used Snyder et al.’s (1991) theoretically derived measure of hope, and showed that parental hope might vary as a function of child characteristics, no measures  of parental psychological outcome were included. |
| McLean & Halstead [50] | Resilience has been defined by Rutter (1987) as being “concerned with individual variations in response to risk. Some people succumb to stress and adversity whereas others overcome life hazards”. | McConnell and Savage (2015) suggested the **ecocultural theory** (Weisner, Matheson, Coots, & Bernheimer,  2005) offers a conceptual approach to investigating the resilience of families of children with ID, as this approach focuses more on the availability of accessibility of culturally relevant resources than individual factors. |
| Miller et al. [51] | No concrete definition for resilience provided | Participants were part of an ongoing longitudinal study of the mechanisms underlying anxiety, avoidance, and arousal in girls with FXS. |
| Raspa et al. [52] | No concrete definition for resilience provided | Mention of the conceptual **model of family adaptation** to FXS |

**Supplementary Material 4B:** Resilience definitions and Models/Framework Used – Intervention studies

| **Reference** | **Resilience Definition** | **Model/Framework** |
| --- | --- | --- |
| **ASD** | | |
| Mackay et al. [53] | “Dynamic process (rather than an outcome) that consists of positive adaptation despite significant adversity” (Herrman et al., 2011; Luthar et al., 2000) | **Resilience framework:** recognizes the adversity facing adolescents with ASD; directs interventions to focus on promoting protective factors that alter the effects of diversity to achieve a positive outcome (Alvord & Grados, 2015; Benzies & Mychasiuk, 2009; Fergus & Zimmerman, 2005; Luthar et al., 2000) |
| Shochet et al. [54] | "Resilience, a dynamic process consisting of positive adaptation despite adversity (Luthar et al., 2000), is inhibited by risk factors, and is promoted by protective factors (Fergus & Zimmerman, 2005), with increased capacity for resilience detected in outcomes such as increased coping self-efficacy" | **Integrated Autism (Autism CRC) Conceptual Model:** designed to promote the mental health of young adolescents on the spectrum (Shochet et al., 2016) by developing, implementing and evaluating a multilevel resilience intervention promoting a greater ability to regulate emotions and improved relationships at the early adolescent, family and school levels.  **Index for Inclusion Framework:** play a critical role in supporting school connectedness and should continue to be implemented in schools. |
| Kuhlthau et al. [55] | “Resiliency is the ability to cope and adapt with ongoing stressors” | No framework or model specified |
| **Learning Disabilities** | | |
| Ahmadi et al. [56] | No definition for resilience provided | **Positive Psychiatry Model:** gratitude, positive emotion, and proactive coping style were reversely associated with the severity of PTSD symptoms (Vernon et al., 2009) |
| Firth et al., [57] | No definition for resilience provided | **Universal Coping Program** (within a model of  “dyslexia-friendly whole-school change”) (Bond et al., 2001; Firth et al., 2008; Greenberg et al., 2003)  Modeled after 3 studies/ programs:  (1) Program for adolescents who had SLD/dyslexia that comprised online awareness and communication skills supported by self advocacy activities at the students’ schools. (Kotzer and Margalit, 2007)  (2) Program of mindfulness and martial arts for adolescents who had SLD/dyslexia was associated with decreased externalizing behaviours and decreased anxiety (Haydicky, 2009).  (3) Program for lower secondary level adolescents who had SLD/dyslexia that taught strategies of challenging negative self-talk, assertiveness training, problem solving and goal setting, all specifically in relation to coping adaptively with dyslexia (Firth, Frydenberg, and Greaves, 2008). |
| **Developmental Disabilities** | | |
| Katz et al. [58] | “Resilience can be defined as the ability to maintain or return to a state of well-being following adverse life events (Dray et al. 2017). Protective factors that have been shown to sup- port resilience include both intrapersonal qualities and skills often referred to as assets and interpersonal factors commonly termed resources” | **Universal Mental Health Literacy Framework:** typically-developing peers are taught methods for providing social supports and being a compassionate friend, alongside students with DD.  Evidence-based practices from the field of special education; included visual supports (Knight et al. 2015; Spriggs et al. 2016), role play (Najdowski et al. 2017), explicit instruction (Knight and Sartini 2014), reinforcement (Odom et al. 2010), and social narratives (Odom et al. 2010). As well, mounting evidence suggests general education content and curricula targeted for typically-developing learners also benefits students with disabilities, including DD, when adapted and modified to meet their needs (Lee et al. 2010). |

1. Bekhet, A.K., N.L. Johnson, and J.A. Zauszniewski, *Effects on resilience of caregivers of persons with autism spectrum disorder: the role of positive cognitions.* Journal of the American Psychiatric Nurses Association, 2012. **18**(6): p. 337-44.

2. Bekhet, A.K., *The Mediating Effects of Positive Cognitions on Autism Caregivers' Depression and Their Children's Challenging Behaviors.* Archives of Psychiatric Nursing, 2016. **30**(1): p. 13-8.

3. Bayat, M., *Evidence of resilience in families of children with autism.* Journal of Intellectual Disability Research, 2007. **51**(Pt 9): p. 702-14.

4. Bitsika, V., D.A. Heyne, and C.F. Sharpley, *The inverse association between psychological resilience and emerging school refusal among bullied autistic youth.* Research in Developmental Disabilities, 2022. **120**: p. 104121.

5. Bitsika, V. and C.F. Sharpley, *Which psychological resilience attributes are associated with lower aspects of anxiety in boys with an autism spectrum disorder? Implications for guidance and counselling interventions.* British Journal of Guidance & Counselling, 2014. **42**(5): p. 544-556.

6. Fong, V., E. Gardiner, and G. Iarocci, *Satisfaction with informal supports predicts resilience in families of children with autism spectrum disorder.* Autism, 2021. **25**(2): p. 452-463.

7. Hayes, K.N., K.G. Rossetti, and K. Zlomke, *Community support, family resilience and mental health among caregivers of youth with autism spectrum disorder.* Child: Care, Health & Development, 2023. **49**(1): p. 130-136.

8. Iannuzzi, D., et al., *Challenges and Growth: Lived Experience of Adolescents and Young Adults (AYA) with a Sibling with ASD.* Journal of Autism & Developmental Disorders, 2022. **52**(6): p. 2430-2437.

9. King, G., et al., *Parent views of the positive contributions of elementary and high school-aged children with autism spectrum disorders and Down syndrome.* Child: Care, Health & Development, 2012. **38**(6): p. 817-28.

10. McCarthy, S.A., et al., *Subclinical autistic traits mediate the relationship between emotional intelligence and resiliency in adolescents.* Personality and Individual Differences Vol 158 2020, ArtID 109845, 2020. **158**.

11. McConnell, D., A. Savage, and R. Breitkreuz, *Resilience in families raising children with disabilities and behavior problems.* Research in Developmental Disabilities, 2014. **35**(4): p. 833-48.

12. McCrimmon, A.W., R.L. Matchullis, and A.A. Altomare, *Resilience and emotional intelligence in children with high-functioning autism spectrum disorder.* Developmental Neurorehabilitation, 2016. **19(3)**: p. 154-161.

13. Menezes, M., et al., *Relations among co-occurring psychopathology in youth with autism spectrum disorder, family resilience, and caregiver coping.* Research in Autism Spectrum Disorders, 2021. **85 (no pagination)**.

14. Montes, G. and J.S. Halterman, *Psychological functioning and coping among mothers of children with autism: A population-based study.* Pediatrics, 2007. **119(5)**: p. e1040-e1046.

15. Nahar, S., Z. Zambelli, and E.J. Halstead, *Risk and protective factors associated with maternal mental health in mothers of children with autism spectrum disorder.* Research in Developmental Disabilities, 2022. **131 (no pagination)**.

16. O'Brien, S., *Families of Adolescents with Autism: Facing the Future.* Journal of Pediatric Nursing, 2016. **31**(2): p. 204-13.

17. Picardi, A., et al., *Parental Burden and its Correlates in Families of Children with Autism Spectrum Disorder: A Multicentre Study with Two Comparison Groups.* Clin Pract Epidemiol Ment Health, 2018. **14**: p. 143-176.

18. Ruiz-Robledillo, N., et al., *Highly resilient coping entails better perceived health, high social support and low morning cortisol levels in parents of children with autism spectrum disorder.* Research in Developmental Disabilities, 2014. **35**(3): p. 686-95.

19. Schneider, M., J. VanOrmer, and K. Zlomke, *Adverse Childhood Experiences and Family Resilience Among Children with Autism Spectrum Disorder and Attention-Deficit/Hyperactivity Disorder.* Journal of Developmental & Behavioral Pediatrics, 2019. **40**(8): p. 573-580.

20. Szatmari, P., et al., *Resilience and developmental health in autism spectrum disorder*, in *Positive mental health, fighting stigma and promoting resiliency for children and adolescents*. 2016, Elsevier Academic Press; US: San Diego, CA. p. 91-109.

21. Tomeny, T.S., *Parenting stress as an indirect pathway to mental health concerns among mothers of children with autism spectrum disorder.* Autism, 2017. **21**(7): p. 907-911.

22. Zhao, M. and W. Fu, *The resilience of parents who have children with autism spectrum disorder in China: a social culture perspective.* International Journal of Developmental Disabilities, 2022. **68(2)**: p. 207-218.

23. Chan, E.S.M., et al., *Are There Resilient Children with ADHD?* Journal of Attention Disorders, 2022. **26**(5): p. 643-655.

24. Dvorsky, M.R., et al., *Coping with COVID-19: Longitudinal Impact of the Pandemic on Adjustment and Links with Coping for Adolescents with and without ADHD.* Research on Child and Adolescent Psychopathology, 2022. **50**(5): p. 605-619.

25. Dvorsky, M.R., et al., *Trajectories of Global Self-Worth in Adolescents with ADHD: Associations with Academic, Emotional, and Social Outcomes.* Journal of Clinical Child & Adolescent Psychology, 2019. **48**(5): p. 765-780.

26. Dvorsky, M.R., et al., *The Protective Effects of Social Factors on the Academic Functioning of Adolescents With ADHD.* Journal of Clinical Child & Adolescent Psychology, 2018. **47**(5): p. 713-726.

27. Mikami, A.Y. and S.P. Hinshaw, *Resilient adolescent adjustment among girls: buffers of childhood peer rejection and attention-deficit/hyperactivity disorder.* Journal of Abnormal Child Psychology, 2006. **34**(6): p. 825-39.

28. Ray, A.R., S.W. Evans, and J.M. Langberg, *Factors Associated with Healthy and Impaired Social Functioning in Young Adolescents with ADHD.* Journal of Abnormal Child Psychology, 2017. **45**(5): p. 883-897.

29. Regalla, M.A., et al., *Attention deficit hyperactivity disorder is an independent risk factor for lower resilience in adolescents: a pilot study.* Trends in Psychiatry & Psychotherapy, 2015. **37**(3): p. 157-60.

30. Regalla, M.A.R., et al., *Resilience levels among adolescents with ADHD using quantitative measures in a family-design study.* Trends in Psychiatry & Psychotherapy, 2019. **41**(3): p. 262-267.

31. Song, J., et al., *Behavioral and mental health problems in adolescents with ADHD: Exploring the role of family resilience.* Journal of Affective Disorders, 2021. **294**: p. 450-458.

32. Uddin, J., et al., *Parenting stress and family resilience affect the association of adverse childhood experiences with children's mental health and attention-deficit/hyperactivity disorder.* Journal of Affective Disorders, 2020. **272**: p. 104-109.

33. Ünver, H., A. Rodopman Arman, and Ş. Nur Akpunar, *Metacognitive Awareness and Emotional Resilience in Children with Attention Deficit Hyperactivity Disorder.* Scand J Child Adolesc Psychiatr Psychol, 2022. **10**(1): p. 33-39.

34. Durish, C.L., K.O. Yeates, and B.L. Brooks, *Psychological Resilience as a Predictor of Symptom Severity in Adolescents With Poor Recovery Following Concussion.* Journal of the International Neuropsychological Society : JINS, 2019. **25(4)**: p. 346-354.

35. Ernst, N., et al., *Lower post-injury psychological resilience is associated with increased recovery time and symptom burden following sport-related concussion.* Applied Neuropsychology. Child, 2022. **11**(4): p. 781-788.

36. Laliberte Durish, C., K.O. Yeates, and B.L. Brooks, *Psychological Resilience as a Predictor of Persistent Post-Concussive Symptoms in Children With Single and Multiple Concussion.* Journal of the International Neuropsychological Society, 2018. **24**(8): p. 759-768.

37. Tonks, J., et al., *Resilience and the mediating effects of executive dysfunction after childhood brain injury: a comparison between children aged 9-15 years with brain injury and non-injured controls.* Brain Injury, 2011. **25**(9): p. 870-81.

38. Greeff, A.P. and C. Nolting, *Resilience in families of children with developmental disabilities.* Families, Systems, & Health, 2013. **31**(4): p. 396-405.

39. Harrowell, I., et al., *Mental health outcomes of developmental coordination disorder in late adolescence.* Developmental Medicine & Child Neurology, 2017. **59**(9): p. 973-979.

40. Janssen, C.G., et al., *Course of health-related quality of life in 9-16-year-old children with cerebral palsy: associations with gross motor abilities and mental health.* Disability & Rehabilitation, 2010. **32**(4): p. 344-51.

41. Lingam, R.P., et al., *The importance of identity and empowerment to teenagers with developmental co-ordination disorder.* Child: Care, Health & Development, 2014. **40**(3): p. 309-18.

42. Schuengel, C., et al., *Self-worth, perceived competence, and behaviour problems in children with cerebral palsy.* Disability & Rehabilitation, 2006. **28**(20): p. 1251-8.

43. Stang, K., et al., *Understanding children with cerebral palsy and bullying: A mixed methods approach.* Child: Care, Health & Development, 2020. **46**(3): p. 303-309.

44. Widyawati, Y., et al., *Positive parenting and its mediating role in the relationship between parental resilience and quality of life in children with developmental disabilities in Java Island, Indonesia.* Research in Developmental Disabilities, 2021. **112 (no pagination)**.

45. Woodman, A.C. and P. Hauser-Cram, *The role of coping strategies in predicting change in parenting efficacy and depressive symptoms among mothers of adolescents with developmental disabilities.* Journal of Intellectual Disability Research, 2013. **57**(6): p. 513-30.

46. Hall, A.M. and L.C. Theron, *Resilience Processes Supporting Adolescents With Intellectual Disability: A Multiple Case Study.* Intellectual & Developmental Disabilities, 2016. **54**(1): p. 45-62.

47. Hauser, C.T., S.T. Kover, and L. Abbeduto, *Maternal well-being and child behavior in families with fragile X syndrome.* Research in Developmental Disabilities, 2014. **35**(10): p. 2477-86.

48. Jordan, T.L., et al., *COVID-19 Pandemic: Mental Health in Girls With and Without Fragile X Syndrome.* Journal of Pediatric Psychology, 2022. **47**(1): p. 25-36.

49. Lloyd, T.J. and R. Hastings, *Hope as a psychological resilience factor in mothers and fathers of children with intellectual disabilities.* Journal of Intellectual Disability Research, 2009. **53(12)**: p. 957-968.

50. McLean, S. and E.J. Halstead, *Resilience and stigma in mothers of children with emotional and behavioural difficulties.* Research in Developmental Disabilities, 2021. **108**: p. 103818.

51. Miller, J.G., et al., *Empathy and Anxiety in Young Girls with Fragile X Syndrome.* Journal of Autism and Developmental Disorders, 2022. **52(5)**: p. 2213-2223.

52. Raspa, M., et al., *Modeling family adaptation to fragile X syndrome.* American Journal on Intellectual & Developmental Disabilities, 2014. **119**(1): p. 33-48.

53. Mackay, B.A., I.M. Shochet, and J.A. Orr, *A Pilot Randomised Controlled Trial of a School-Based Resilience Intervention to Prevent Depressive Symptoms for Young Adolescents with Autism Spectrum Disorder: A Mixed Methods Analysis.* J Autism Dev Disord, 2017. **47**(11): p. 3458-3478.

54. Shochet, I.M., et al., *A school-based approach to building resilience and mental health among adolescents on the autism spectrum: A longitudinal mixed methods study.* School Mental Health: A Multidisciplinary Research and Practice Journal, 2022: p. No Pagination Specified.

55. Kuhlthau, K.A., et al., *Resiliency Intervention for Siblings of Children With Autism Spectrum Disorder: A Randomized Pilot Trial.* Academic Pediatrics., 2023.

56. Ahmadi, N., et al., *A Randomized Controlled Feasibility Trial of Reminder-Focused Positive Psychiatry in Adolescents With Comorbid Attention-Deficit/Hyperactivity Disorder and Posttraumatic Stress Disorder.* Prim Care Companion CNS Disord, 2020. **22**(5).

57. Firth, N., et al., *Coping successfully with dyslexia: an initial study of an inclusive school-based resilience programme.* Dyslexia, 2013. **19**(2): p. 113-30.

58. Katz, J., et al., *Effects of a Universal School-Based Mental Health Program on the Self-concept, Coping Skills, and Perceptions of Social Support of Students with Developmental Disabilities.* J Autism Dev Disord, 2020. **50**(11): p. 4069-4084.
