# Supplementary material for: Resilience and adolescence-transition in youth with developmental disabilities and their families: a scoping review
Source: Front Rehabil Sci. 2024 Feb 27;5:1341740. doi: 10.3389/fresc.2024.1341740 (PMC10927845; doi:10.3389/fresc.2024.1341740)
Supplement: Supplementary file 5 [file Table5.docx]

**Supplementary Material 5** Coded resilience factors in observational studies

| **Publication** | | | **All resilience factors addressed at large in observational studies (n=53)** | | | |  | **Factors determined to directly affect resilience among ADOLESCENTS** | | | |  | **Factors determined to directly affect resilience among CAREGIVERS/ SIBLINGS** | | | |
| --- | --- | --- | --- | --- | --- | --- | --- | --- | --- | --- | --- | --- | --- | --- | --- | --- |
|  | **First Author** | **Year** | **A Factors**  **Individual** | **B Factors**  **Family** | **C Factors**  **School-peers** | **D Factors**  **Community** |  | **A Factors**  **Individual** | **B Factors**  **Family** | **C Factors**  **School-peers** | **D Factors**  **Community** |  | **A Factors**  **Individual** | **B Factors**  **Family** | **C Factors**  **School-peers** | **D Factors**  **Community** |
| 1 | Bekhet | 2012 | 12, 16, 27 | 2 | 0 | 2 |  | 0 | 0 | 0 | 0 |  | **12, 16** | 0 | 0 | 0 |
| 2 | Bekhet | 2016 | 2, 12, 19, 24 | 2, 3, 4 | 0 | 0 |  | **19** | 0 | 0 | 0 |  | **2, 12** | **4** | 0 | 0 |
| 3 | Bayat | 2007 | 5, 11, 16, 17, 18, 23, 24, 25, 26 | 1, 2, 3 | 3, 4 | 1, 2 |  | 16 | 0 | 0 | 0 |  | **16, 18, 23, 24, 25, 26** | **1, 2, 3** | 0 | **1, 2** |
| 5 | Bitsika | 2022 | 2, 3, 4, 8, 12, 15, 16, 17, 19, 22, 24, 25, 26, 27, 28 | 1 | 1, 2, 4, 5 | 1 |  | **12, 16, 17, 19, 22, 27, 28** | 0 | **1, 2, 4** | 0 |  | 0 | 0 | 0 | 0 |
| 6 | Bitsika | 2014 | 1, 2, 4, 7, 8, 10, 12, 16, 17, 19, 27, 28 | 0 | 1, 5 | 0 |  | **2, 8, 12, 16, 17, 27, 28** | 0 | **1, 5** | 0 |  | 0 | 0 | 0 | 0 |
| 7 | Chan | 2022 | 2, 19, 22, 27, 28 | 1, 2, 3, 4, 5 | 4 | 2, 6 |  | **2, 19, 22** | 0 | 0 | 0 |  | 0 | 0 | 0 | 0 |
| 8 | Durish | 2019 | 2, 4, 16, 19, 23, 24 | 3 | 0 | 0 |  | **2, 16, 19, 23, 24** | 0 | 0 | 0 |  | 0 | 0 | 0 | 0 |
| 9 | Dvorsky | 2022 | 1, 2, 4, 7, 17, 20, 21, 22, 26, 28 | 1, 2, 3, 4 | 1, 2, 3, 4, 5 | 1, 2, 3, 4 |  | **2, 4, 7, 17, 20, 21, 22, 26, 28** | **3** | **4** | **3, 4** |  | 0 | 0 | 0 | 0 |
| 10 | Dvorsky | 2019 | 2, 3, 4, 8, 11, 16, 19, 20, 21, 26, 27, 28 | 1, 3 | 1, 2, 3, 4, 5 | 3 |  | **2, 16, 19, 20, 21, 26, 27, 28** | 0 | **1, 5** | 0 |  | 0 | 0 | 0 | 0 |
| 11 | Dvorsky | 2018 | 3, 8, 15, 16, 19, 26, 27, 28 | 3, 4 | 1, 2, 3, 4, 5 | 3 |  | **26, 27, 28** | 0 | **1, 2, 3, 4, 5** | 0 |  | 0 | 0 | 0 | 0 |
| 12 | Ernst | 2022 | 2, 4, 7, 12, 16, 23, 24, 26, 27 | 1, 2 | 1,2 | 0 |  | **7, 12, 16, 27** | 0 | 0 | 0 |  | 0 | 0 | 0 | 0 |
| 13 | Fong | 2021 | 3, 4, 16, 17, 18, 26, 27, 28 | 1, 2, 3 | 1, 2 | 1, 2, 3, 5 |  | 0 | 0 | 0 | 0 |  | **26, 27, 28** | **1, 2, 3** | **1, 2** | **1, 2, 3, 5** |
| 14 | Greeff | 2013 | 3, 4, 6, 9, 11, 16, 17, 18, 19, 26, 27 | 1, 2, 3, 4, 5 | 1, 2, 3, 6 | 1, 2, 3, 6 |  | 0 | 0 | 0 | 0 |  | **3, 4, 9, 16, 17, 26, 27** | **1, 2, 3** | 0 | 0 |
| 15 | Hall | 2016 | 3, 4, 6, 7, 11, 12, 13, 15, 16, 17, 18, 19, 20, 21, 22, 23, 24, 25, 26, 27, 28 | 1, 2, 3, 5 | 1, 2, 3, 4, 5, 6 | 1, 2, 3, 5, 6 |  | **3, 6, 7, 12, 13, 19, 20, 21, 22, 23, 24, 25, 26, 27, 28** | **1, 2, 3, 5** | **1, 2, 3, 4, 5, 6** | **1, 2, 3, 5, 6** |  | 0 | 0 | 0 | 0 |
| 16 | Harrowell | 2017 | 3, 7, 16, 17, 21, 22, 24, 27, 28 | 1, 2, 3, 4 | 1, 2, 5 | 1, 5 |  | **3, 7, 16, 17, 21, 22, 24, 27, 28** | 0 | 0 | 0 |  | 0 | 0 | 0 | 0 |
| 17 | Hauser | 2014 | 3, 4, 7, 10, 16, 22, 23, 27 | 1, 2, 3, 4 | 4 | 0 |  | 0 | 0 | 0 | 0 |  | **16, 22, 23, 27** | **1, 2, 3, 4** | 0 | 0 |
| 18 | Hayes | 2023 | 3, 4, 13, 16, 17, 26, 27 | 1, 2, 3, 4 | 0 | 1, 2, 3 |  | 0 | 0 | 0 | 0 |  | **3, 16, 17, 26, 27** | **1, 2, 3, 4** | 0 | **1, 2, 3** |
| 19 | Iannuzzi | 2022 | 3, 4, 7, 8, 12, 21, 22, 24, 25, 26, 27, 28 | 1, 2, 3 | 1, 2, 5 | 1, 2, 5 |  | 0 | 0 | 0 | 0 |  | **7, 8, 12, 22, 24, 25, 26, 27** | **1, 2, 3** | **1, 2** | 0 |
| 20 | Janssen | 2010 | 1, 2, 3, 16, 17, 27, 28 | 1, 2, 3 | 1, 2, 5 | 0 |  | **1, 2, 16, 17, 27, 28** | 0 | **1, 2, 5** | 0 |  | 0 | 0 | 0 | 0 |
| 21 | Jordan | 2022 | 3, 4, 7, 10, 16, 22, 23, 26, 27, 28 | 1, 2, 3, 4 | 1, 2, 3, 4, 5 | 1, 2, 3, 4, 5 |  | **3, 4, 7, 16, 22, 23, 26, 27, 28** | **1, 2** | **1, 2, 5** | **1, 4** |  | 0 | 0 | 0 | 0 |
| 22 | King | 2012 | 4, 5, 9, 13, 16, 17, 18, 24, 25, 26 | 1, 2, 3, 4 | 1, 2 | 1, 2, 3, 5 |  | 0 | 0 | 0 | 0 |  | **9, 13, 16, 17, 25, 26** | **1, 2, 3, 4** | 0 | **1, 2, 3, 5** |
| 23 | Laliberté-Durish | 2018 | 2, 7, 16, 17, 18, 27 | 3 | 0 | 0 |  | **2, 7, 16, 17, 27** | 0 | 0 | 0 |  | 0 | 0 | 0 | 0 |
| 24 | Lingam | 2013 | 1, 3, 4, 5, 6, 9, 11, 16, 17, 21, 24, 25, 27 | 1, 2, 3, 5 | 1, 2, 3, 4, 5, 6 | 2, 5, 6 |  | **4, 9, 11, 16, 21, 25** | 0 | 0 | **2** |  | 0 | 0 | 0 | 0 |
| 25 | Lloyd | 2009 | 4, 11, 12, 13, 15, 16, 17, 19, 20, 21, 22, 23, 24, 25, 26 | 1, 2, 3, 4 | 0 | 0 |  | 0 | 0 | 0 | 0 |  | **4, 12, 13, 16, 17, 19, 20, 21, 22, 23, 24, 25** | **1** | 0 | 0 |
| 26 | McCarthy | 2020 | 2, 3, 7, 8, 16, 17, 19, 20, 22, 27, 28 | 2, 3 | 1, 2 | 1, 5 |  | **2, 3, 7, 16, 17, 19, 20, 22, 27, 28** | **2** | **1** | **1** |  | 0 | 0 | 0 | 0 |
| 27 | McConnell | 2014 | 3, 4, 6, 11, 16, 25, 27 | 1, 2, 3, 4, 5 | 1, 2, 6 | 1, 2, 3, 4, 5, 6 |  | 0 | 0 | 0 | 0 |  | **3, 6, 27** | **1, 2, 3, 5** | **1, 2, 6** | **1, 2, 3, 4, 5, 6** |
| 28 | McCrimmon | 2014 | 2, 7, 8, 16, 17, 19, 20, 21, 22, 23, 27, 28 | 1, 2, 3 | 1, 2 | 1 |  | **2, 7, 8, 16, 17, 19, 20, 21, 22, 23, 27, 28** | **1, 2** | **1, 2** | **1** |  | 0 | 0 | 0 | 0 |
| 29 | McLean | 2021 | 3, 4, 6, 7, 8, 21, 25, 27 | 1, 2, 3, 4, 5 | 1, 2, 6 | 1, 2, 5, 6 |  | 0 | 0 | 0 | 0 |  | **4, 7, 27** | **1, 2, 4, 5** | **2, 6** | **2, 5, 6** |
| 30 | Menezes | 2021 | 2, 4, 13, 17, 24, 27 | 1, 2, 3, 4 | 1, 2 | 2 |  | 0 | 0 | 0 | 0 |  | **4, 17, 24 27** | **1, 2, 3, 4** | 0 | 0 |
| 31 | Mikami | 2006 | 4, 7, 11, 17, 21, 23, 26, 27 | 2 | 1, 2, 3, 4, 5 | 1, 5 |  | **4, 7, 17, 21, 23, 26, 27** | **2** | **1, 2, 4, 5** | **1, 5** |  | 0 | 0 | 0 | 0 |
| 32 | Miller | 2021 | 2, 3, 4, 7, 8, 10, 22, 27, 28 | 3 | 1, 4 | 1 |  | **2, 7, 8, 22, 27, 28** | 0 | **1** | **1** |  | 0 | 0 | 0 | 0 |
| 33 | Montes | 2007 | 3, 4, 7, 16, 23, 26, 27, 28 | 1, 2, 3, 4 | 1, 2 | 1, 2 |  | 0 | 0 | 0 | 0 |  | **3, 4, 7, 26, 27, 28** | **1, 2, 3, 4** | **1, 2** | **1, 2** |
| 34 | Nahar | 2022 | 3, 4, 7, 12, 13, 16, 17, 22, 26, 27 | 1, 2, 3, 4 | 1, 2 | 1, 2 |  | 0 | 0 | 0 | 0 |  | **4, 7, 17, 22, 26, 27** | **1, 2, 3** | 0 | 0 |
| 35 | O'Brien | 2016 | 4, 17, 18, 25, 27 | 1, 2, 3, 4, 5 | 1, 2 | 1, 2, 3 |  | 0 | 0 | 0 | 0 |  | **4, 17, 27** | **3** | 0 | 0 |
| 36 | Picardi | 2018 | 2, 3, 4, 5, 7, 13, 16, 17, 18, 19, 22, 26, 27, 28 | 1, 2, 3, 4 | 1, 2 | 1, 2, 6 |  | 0 | 0 | 0 | 0 |  | **2, 4, 7, 16, 17, 19, 22, 26, 27, 28** | **1, 2** | **1, 2** | **1, 2** |
| 37 | Raspa | 2014 | 4, 5, 9, 10, 13, 16, 20, 26, 27 | 1, 2, 3, 4 | 1, 2 | 1, 2 |  | 0 | 0 | 0 | 0 |  | **4, 26, 27** | **1, 2, 3, 4** | **1, 2** | **1, 2** |
| 38 | Ray | 2016 | 3, 8, 16, 19, 23, 25, 26, 27, 28 | 1, 2, 3, 4 | 1, 2, 4, 5 | 1, 2, 3, 5 |  | **23, 23, 26, 27, 28** | **1, 2, 3, 4** | **1, 2, 4, 5** | **1, 2, 3, 5** |  | 0 | 0 | 0 | 0 |
| 39 | Regalla | 2015 | 1, 5, 16, 21, 23, 25 | 3, 4 | 0 | 0 |  | **16, 21, 23** | **3, 4** | 0 | 0 |  | 0 | 0 | 0 | 0 |
| 40 | Regalla | 2019 | 2, 10, 26, 27 | 1, 2, 3, 4 | 4 | 0 |  | **27** | **1, 2** | 0 | 0 |  | 0 | 0 | 0 | 0 |
| 41 | Ruiz-Robledillo | 2014 | 1, 4, 10, 16, 19, 22, 23, 27 | 1, 2, 3 | 1, 2 | 1, 2 |  | 0 | 0 | 0 | 0 |  | **10, 16, 19, 22, 23, 27** | **1, 2, 3** | **1, 2** | **1, 2** |
| 42 | Schneider | 2019 | 3, 4, 13, 16, 17, 27 | 1, 2, 3, 4 | 1, 2 | 1, 2, 3 |  | 0 | 0 | 0 | 0 |  | **4, 13, 16, 17, 27** | **1, 2, 3, 4** | **1, 2** | **1, 2, 3** |
| 43 | Schuengel | 2006 | 1, 2, 16, 21, 23, 24, 25, 26, 27 | 1, 2, 3 | 1, 2 | 0 |  | **2, 16, 21, 23, 24, 25, 26, 27** | **2** | 0 | 0 |  | 0 | 0 | 0 | 0 |
| 44 | Song | 2021 | 3, 4, 5, 7, 13, 16, 17, 19, 21, 22, 26, 27, 28 | 1, 2, 3, 4 | 1, 2, 4 | 2 |  | 0 | 0 | 0 | 0 |  | **4, 7, 16, 19, 21, 22, 26, 27, 28** | **1, 2, 3, 4** | 0 | 0 |
| 45 | Stang | 2020 | 2, 4, 5, 6, 17, 21, 23, 24, 25, 27, 28 | 2 | 1, 2, 3, 4, 5 | 1, 2, 3, 4, 5 |  | **5, 21, 23, 24, 25, 27, 28** | **2** | **1, 2, 3, 4, 5** | **1, 2, 3, 5** |  | 0 | 0 | 0 | 0 |
| 46 | Szatmari | 2016 | 1, 2, 3, 4, 6, 7, 9, 11, 12, 15, 16, 17, 18, 19, 20, 22, 26, 27, 28 | 1, 2, 3, 4, 5 | 1, 2, 3, 4, 5, 6 | 1, 2, 3, 4, 5, 6 |  | **2, 3, 4, 26, 27** | **1, 2, 3, 4** | **1, 2, 3, 4, 5** | **1, 2, 3, 4, 5** |  | 0 | 0 | 0 | 0 |
| 47 | Tomeny | 2016 | 4, 10, 19, 22, 23, 25, 26, 27, 28 | 1, 2, 3, 4 | 1 | 0 |  | 0 | 0 | 0 | 0 |  | **4, 19, 22, 23, 25, 26, 27, 28** | 0 | 0 | 0 |
| 48 | Tonks | 2011 | 2, 4, 7, 8, 15, 16, 17, 19, 20, 22, 27, 28 | 1, 2, 3 | 1, 4 | 0 |  | **2, 4, 7, 16, 17, 19, 20, 22, 27, 28** | 0 | 0 | 0 |  | 0 | 0 | 0 | 0 |
| 49 | Uddin | 2020 | 7, 13, 16, 17, 19, 22, 26, 27 | 1, 2, 3, 4 | 1, 2, 4 | 1, 2, 3, 5, 6 |  | **7, 16, 17, 19, 22, 26, 27** | **1, 2, 3, 4** | 0 | **1, 2, 3, 4, 5, 6** |  | **7, 16, 17, 19, 22, 26, 27** | **1, 2, 3, 4** | 0 | **1, 2, 3, 5, 6** |
| 50 | Ünver | 2022 | 2, 4, 7, 12, 15, 16, 17, 19, 22, 23, 24, 27, 28 | 1, 2, 3 | 1, 2, 3, 4, 5 | 1, 5 |  | **2, 4, 7, 12, 16, 17, 19, 22, 23, 24, 27, 28** | 0 | 0 | 0 |  | 0 | 0 | 0 | 0 |
| 51 | Widyawati | 2021 | 3, 4, 5, 7, 16, 17, 19, 21, 27, 28 | 1, 2, 3, 4, 5 | 2, 6 | 2, 3, 5, 6 |  | 0 | 0 | 0 | 0 |  | **4, 7, 16, 17, 19, 27, 28** | **1, 2, 3, 4** | **2** | **2, 3** |
| 52 | Woodman | 2012 | 1, 2, 4, 5, 7, 12, 16, 17, 20, 22, 27, 28 | 1, 2, 3, 4 | 0 | 0 |  | 0 | 0 | 0 | 0 |  | **2, 4, 5, 7, 16, 17, 20, 22, 27, 28** | **1, 2, 3, 4** | 0 | 0 |
| 53 | Zhao | 2020 | 3, 4, 6, 7, 13, 14, 16, 17, 20, 23, 25, 26, 27, 28 | 1, 2, 3, 4, 5 | 1, 2, 3, 4, 5 | 1, 2, 3, 4, 5, 6 |  | 0 | 0 | 0 | 0 |  | **3, 4, 6, 16, 20, 23, 25, 26, 27, 28** | **1, 2, 3, 4, 5** | **1, 6** | **1, 2, 3, 4, 6** |

Legend: A factors refer to Individual resilience factors; B Factors refer to Family/Home resilience factors; C Factors refer to Schools/Peers resilience factors; D Factors refer to Community resilience factors. Refer to Supplementary material 2 for more information about the coding structure.
